# EF-P Posttranslational Modification Has Variable Impact on Polyproline Translation in *Bacillus subtilis*

**DOI:** 10.1128/mBio.00306-18

**Published:** 2018-04-03

**Authors:** Anne Witzky, Katherine R. Hummels, Rodney Tollerson, Andrei Rajkovic, Lisa A. Jones, Daniel B. Kearns, Michael Ibba

**Affiliations:** a Department of Molecular Genetics, Ohio State University, Columbus, Ohio, USA; b Center for RNA Biology, Ohio State University, Columbus, Ohio, USA; c Department of Biology, Indiana University, Bloomington, Indiana, USA; d Department of Microbiology, Ohio State University, Columbus, Ohio, USA; e Proteomics Facility, Fred Hutchinson Cancer Research Center, Seattle, Washington, USA; National Cancer Institute

**Keywords:** elongation, posttranslational modification, protein synthesis, translational

## Abstract

Elongation factor P (EF-P) is a ubiquitous translation factor that facilitates translation of polyproline motifs. In order to perform this function, EF-P generally requires posttranslational modification (PTM) on a conserved residue. Although the position of the modification is highly conserved, the structure can vary widely between organisms. In *Bacillus subtilis*, EF-P is modified at Lys32 with a 5-aminopentanol moiety. Here, we use a forward genetic screen to identify genes involved in 5-aminopentanolylation. Tandem mass spectrometry analysis of the PTM mutant strains indicated that *ynbB*, *gsaB*, and *ymfI* are required for modification and that *yaaO*, *yfkA*, and *ywlG* influence the level of modification. Structural analyses also showed that EF-P can retain unique intermediate modifications, suggesting that 5-aminopentanol is likely directly assembled on EF-P through a novel modification pathway. Phenotypic characterization of these PTM mutants showed that each mutant does not strictly phenocopy the *efp* mutant, as has previously been observed in other organisms. Rather, each mutant displays phenotypic characteristics consistent with those of either the *efp* mutant or wild-type *B. subtilis* depending on the growth condition. *In vivo* polyproline reporter data indicate that the observed phenotypic differences result from variation in both the severity of polyproline translation defects and altered EF-P context dependence in each mutant. Together, these findings establish a new EF-P PTM pathway and also highlight a unique relationship between EF-P modification and polyproline context dependence.

## IMPORTANCE

Despite the high level of conservation of EF-P, the posttranslational modification pathway that activates EF-P is highly divergent between species. Here, we have identified and characterized in *B. subtilis* a novel posttranslational modification pathway. This pathway not only broadens the scope of potential EF-P modification strategies, but it also indicates that EF-P modifications can be assembled directly on EF-P. Furthermore, characterization of these PTM mutants has established that an altered modification state can impact both the severity of polyproline translational defects and context dependence.

## INTRODUCTION

During translation, the ribosome employs aminoacyl-tRNAs and a number of translation factors in order to decode an mRNA and synthesize a polypeptide. The rate of translation can depend on a number of factors such as codon usage, mRNA structure, and amino acid structure (1, 2). For example, proline has a unique pyrrolidine ring structure that creates significant steric constraints, making proline both a poor peptidyl acceptor and donor (3). Due to this limitation, translation of polyproline can be substantially slower than translation of other amino acid motifs and often results in translational pausing (4, 5). In order to alleviate polyproline-induced translational pausing, a universally conserved translation factor, elongation factor P (EF-P) (eukaryotic initiation factor 5A [eIF5A] in eukaryotes and archaeal initiation factor 5A [aIF5A] in archaea) binds the ribosome between the P and E sites and entropically stimulates peptide bond formation (6–11).

In order to stimulate translation of polyproline motifs, EF-P requires posttranslational modification (PTM) at a highly conserved residue (12, 13). The structure of the modification can vary substantially between organisms. In eukaryotes, deoxyhypusine synthase (DHS) and deoxyhypusine hydroxylase (DOHH) coordinately modify the EF-P homolog eIF5A with a hypusine moiety, and hypusinated eIF5A is essential for viability (14). In *Pseudomonas aeruginosa* and *Neisseria meningitidis*, EF-P is modified by EarP with a rhamnose moiety. Loss of EF-P or EarP can cause severe growth defects or, in some cases, lethality (15–17). In *Escherichia coli* and *Salmonella enterica*, EpmA, EpmB, and EpmC modify EF-P with *R*-β-lysine (18–20). Although *R*-β-lysylated EF-P is not required for viability, mutants display a wide range of pleiotropic phenotypic characteristics, including growth defects and loss of virulence (20, 21). Recently, the search for EF-P PTMs has been expanded to Gram-positive bacteria. In *Bacillus subtilis*, EF-P is modified with a 5-aminopentanol moiety at Lys32 (22). However, in *B. subtilis*, loss of EF-P does not result in the severe viability and growth defects observed in other bacteria. Instead, the major requirement for EF-P in *B. subtilis* is for swarming motility (22, 23).

We have recently shown that YmfI reduces 5-aminopentanone to 5-aminopentanol in the final step of EF-P modification (24). Further, we showed that 5-aminopentanone, but not unmodified EF-P, is inhibitory to swarming motility, as abolishing posttranslational modification of EF-P altogether by mutation of Lys32 to an arginine suppressed the *ymfI* swarming defect (24). Here, we take advantage of EF-P activity in the absence of modification to identify other enzymes that act upstream of YmfI in the EF-P modification pathway (24). Mass spectrometry analyses of EF-P purified from wild-type (WT) *B. subtilis* and each of the modification mutants revealed that EF-P can retain incomplete modifications. In order to investigate the physiological consequences of producing EF-P with an intermediate modification, we phenotypically characterized these mutants for swarming proficiency and antibiotic resistance. By using this screen, we observed that PTM mutants display phenotypic characteristics similar to those of an *efp* mutant under some growth conditions, but not others. *In vivo* polyproline reporter data revealed that this phenotypic discrepancy is due to variability in polyproline translation defect severity and context dependence in each mutant. Together, these results establish the 5-aminopentanol modification pathway and reveal a relationship between EF-P modification and motif-specific EF-P dependence.

## RESULTS

### A forward genetic screen to identify genes required for modification of EF-P.

We have previously shown that, in the absence of YmfI, EF-P accumulates an intermediate modification on Lys32 that is inhibitory for swarming motility (24). One way to bypass this inhibition was to prevent posttranslational modification of EF-P through mutation of the modification site, Lys32, to an arginine (24). We hypothesized that another way to bypass inhibition was to abolish posttranslational modification of EF-P through deletion of enzymes required for modification. A nonswarming *ymfI efp* (*efp*^+^) sensitized mutant background was transposon mutagenized in 26 parallel replicates, approximately 10,000 colonies were separately combined to form each pool, and the 26 separate pools were used to inoculate swarm agar. After a prolonged lag period, a subpopulation of cells were able to move out from the site of inoculation of each pool. One swarming proficient clone from each pool was isolated and backcrossed to the *ymfI efp* (*efp*^+^) parent by SPP1-mediated phage transduction. Each transposon insertion improved swarming motility, suggesting that the transposon was linked to the phenotype and suppressed the swarming defect of the *ymfI* mutation alone (see Fig. S1A in the supplemental material). The locations of the transposons were determined by inverse PCR.

10.1128/mBio.00306-18.1 FIG S1  Quantitative swarm expansion assay in which mid-log-phase cultures were used to inoculate swarm plates. The swarm radius was monitored along the same axis every 30 min for 6 h. Data points represent the averages of three technical replicates. For panel B, the swarm plates were supplemented with 1 mM IPTG. The strains with the indicated genotype were used as the inoculum in the different panels: (A) *ymfI efp* (*efp*) (DK3789), *ymfI efp* (*efp*) *gsaB*::Tn (DK4304), *ymfI efp* (*efp*) *yaaO*::Tn (DK300), *ymfI efp* (*efp*) *ynbB*::Tn (DK4301), *ymfI efp* (*efp*) *yfkA*::Tn (DK4303), and *ymfI efp* (*efp*) *ywlG*::Tn (DK4368); (B) *yaaO ymfI* (DK4077) and *yaaO ymfI P_xpcA_-yaaO* (DK5305); (C) *yaaO* (DK3894), and *yaaO* (*yaaO*) (DK5327); (D) *yfkA* (DK4564) and *yfkA* (*yfkA*) (DK5165); (E) *ywlG* (DK4612) and *ywlG* (*ywlG*) (DK5166). Download FIG S1, PDF file, 0.1 MB. Copyright © 2018 Witzky et al. 2018 Witzky et al. This content is distributed under the terms of the Creative Commons Attribution 4.0 International license.

**FIG 1:**
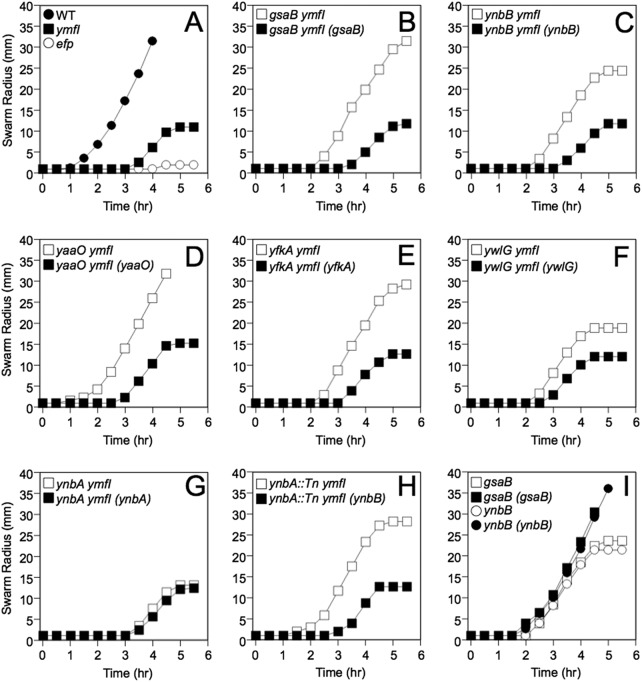
Deletion of *gsaB*, *ynbB*, *yaaO*, *yfkA*, or *ywlG* suppresses the swarming defect of a *ymfI* mutant. A quantitative swarm expansion assay was used in which mid-log-phase cultures were used to inoculate swarm plates. The swarm radius was monitored along the same axis every 30 min for 6 h. Data points represent the average values for three technical replicates. In panel D, swarm plates were supplemented with 1 mM IPTG. The strains with the indicated genotype were used as the inoculum in the different panels: (A) WT (DK1042), *efp* (DK2050), and *ymfI* (DK3621); (B) *gsaB ymfI* (DK5171) and *gsaB ymfI* (*gsaB*) (DK5320); (C) *ynbB ymfI* (DK5172) and *ynbB ymfI* (*ynbB*) (DK5321); (D) *yaaO ymfI* (DK4077) and *yaaO ymfI* (*yaaO*) (DK5328); (E) *yfkA ymfI* (DK5170) and *yfkA ymfI* (*yfkA*) (DK5304); (F) *ywlG ymfI* (DK5174) and *ywlG ymfI* (*ywlG*) (DK5326); (G) *ynbA ymfI* (DK5173) and *ynbA ymfI* (*ynbA*) (DK5322); (H) *ynbA*::Tn *ymfI* (DK5339) and *ynbA*::Tn *ymfI* (*ynbB*) (DK5340); (I) *gsaB* (DK4601), *gsaB* (*gsaB*) (DK5308), *ynbB* (DK4604), and *ynbB* (*ynbB*) (DK5309).

Of the 26 transposon insertion suppressors of *ymfI*, 9 had a mutation in *gsaB*, 8 had a mutation in the *ynbAB* operon, 7 had a mutation in *yaaO*, 1 had a mutation in *yfkA*, and 1 had a mutation in *ywlG* (Table S1). To determine whether each gene disrupted by the transposon insertions was directly responsible for the phenotype, *gsaB*, *ynbA*, *ynbB*, *yaaO*, *yfkA*, and *ywlG*, were disrupted by in-frame markerless deletion. Deletion of *gsaB*, *ynbB*, *yaaO*, *yfkA*, and *ylwG* increased swarming of the *ymfI* mutant, but deletion of *ynbA* did not (Fig. 1A to G). The *gsaB*, *ynbB*, *yfkA*, and *ywlG* deletion mutants could each be complemented by integrating a construct containing the corresponding gene cloned downstream of the putative native promoter at the ectopic *amyE* site in the chromosome (Fig. 1A to C, E, and F). While the *yaaO* deletion mutant could not be complemented by cloning *yaaO* expressed from its putative native promoter, it was complemented when *yaaO* was expressed under the control of an isopropyl-β-d-thiogalactopyranoside (IPTG)-inducible promoter (Fig. S1B and Fig. 1D). We infer that suppression of the *ymfI* swarming defect was caused by deletion of *gsaB*, *ynbB*, *yaaO*, *yfkA*, and *ywlG* rather than polar effects caused by the transposon insertion. In contrast, transposon insertions in *ynbA* were likely polar on *ynbB*, as swarming motility inhibition was restored to the transposon mutants when the *ynbB* complementation construct was ectopically integrated (Fig. 1H). We conclude that deletion of *gsaB*, *yaaO*, *ynbB*, *yfkA*, or *ywlG*, but not *ynbA*, suppresses the *ymfI* mutant swarming defect.

10.1128/mBio.00306-18.6 TABLE S1  Transposon insertion suppressors of *ymfI*. Download TABLE S1, PDF file, 0.1 MB. Copyright © 2018 Witzky et al. 2018 Witzky et al. This content is distributed under the terms of the Creative Commons Attribution 4.0 International license.

**FIG 2:**
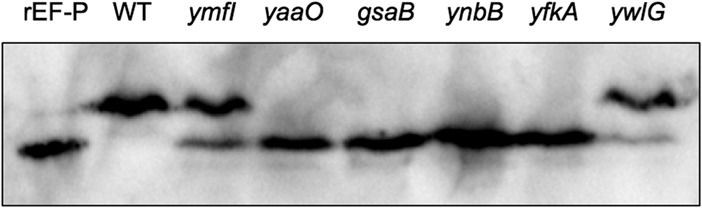
Lysates from WT (DK1042), *ymfI* (DK3621), *yaaO* (DK3894), *gsaB* (DK4601), *ynbB* (DK4604), *yfkA* (DK4564), and *ywlG* (DK4612) strains were resolved via isoelectric focusing. Purified recombinant EF-P (rEF-P) was run as an unmodified control. Blots were probed with anti-EF-P polyclonal antisera.

To determine whether the identified genes impact posttranslational modification of EF-P, each single deletion strain was screened for EF-P electrophoretic mobility by isoelectric focusing and seminative gel electrophoresis. In isoelectric focusing, EF-P in wild-type (WT) *B. subtilis* cells resolved as one band. EF-P in the *yfkA*, *ynbB*, *gsaB*, and *yaaO* mutants migrated as one band with an isoelectric focusing point lower than that of wild-type EF-P, suggesting an alteration in modification state (Fig. 2). Unlike the other mutants, EF-P from the *ywlG* and *ymfI* mutants migrated as two bands (Fig. 2). In seminative gel electrophoresis, EF-P resolved as two bands in the wild-type background (Fig. S2). In each of the mutant strains, except the *ywlG* mutant, EF-P resolved as one band with an electrophoretic mobility similar to that of the lower band in the wild-type strain (Fig. S2). We infer that mutations that restore swarming in the absence of YmfI alter EF-P electrophoretic mobility and may do so by different mechanisms.

10.1128/mBio.00306-18.2 FIG S2  Lysates from WT (DK1042), *ymfI* (DK3621), *gsaB* (DK4554), *yaaO* (DK4325), *ynbB* (DK4389), *yfkA* (DK4555), or *ywlG* (DK4556) were resolved by seminative (top) or denaturing (middle and bottom) polyacrylamide gel electrophoresis and probed with anti-EF-P (top and middle) or anti-SigA (bottom) polyclonal antisera. Download FIG S2, PDF file, 0.03 MB. Copyright © 2018 Witzky et al. 2018 Witzky et al. This content is distributed under the terms of the Creative Commons Attribution 4.0 International license.

### Mutants that suppress the absence of YmfI display aberrant 5-aminopentanolylation.

In order to determine the modification status of EF-P in the absence of GsaB, YaaO, YnbB, YfkA, or YwlG, FLAG-tagged EF-P (EF-P-FLAG) was overexpressed and affinity tag purified from each mutant background, in-gel digested with trypsin, and then resolved in an Orbitrap Elite or Orbitrap Fusion mass spectrometer. In tandem mass spectrometry (MS/MS) analyses, the most abundant ions were selected for electron transfer dissociation (ETD). In the wild-type control, ions corresponding to unmodified EF-P (*m/z* = 314.181; *z* = 4+), as well as 5-aminopentanolylated EF-P (*m/z* = 489.785; *z* = 4+), with an additional mass of 101.084 Da on Lys32 were identified (Table 1 and Fig. S3A and B). Furthermore, additional masses on Lys32 of 82.042 (C_5_H_7_O) and 100.052 (C_5_H_9_O_2_) were also identified in the wild-type strain (Table 1 and Fig. S3C and D). Given the structure of 5-aminopentanol and its precursor, 5-aminopentanone, it is likely that the additional mass of 82.042 corresponds to pentenone and that 100.052 corresponds to hydroxypentanone.

10.1128/mBio.00306-18.3 FIG S3  MS/MS spectra of EF-P peptide containing Lys32. EF-P was purified from strains DK2448 (A to D), DK4310 (E and F), DK4313 (G and H), DK3908 (J to L), DK4572 (M and O), and DK4573 (P to R). Following purification, protein was digested on the gel with trypsin, and then resolved with tandem mass spectrometry on a Orbitrap Elite mass spectrometer (A to H and M to R) or Orbitrap Fusion mass spectrometer (J to L). Modification at Lys32 inhibits trypsin cleavage, resulting in differences in digested peptide products depending on the modification state of Lys32. The parent ions are *z* = 4+ (A to N and P and Q) or *z* = 3+ (O and R). MS/MS for unmodified peptide (A, E, G, J, M, and P; *m/z* = 314.1809) or peptide modified with 5-aminopentanol (B, K, N, and Q; *m/z* = 489.7853), pentenone (C; *m/z* = 485.0248), hydroxypentanone (D; *m/z* = 489.5274), or an acetylation (F, H, L, O, and R; *m/z* = 475.0169 and *z* = 4+ or *m/z* = 633.0200 and *z* = 3+) are shown. Modifications on Lys32 are marked as follows: AP, 5-aminopentanol; P, pentenone; HP, hydroxypentanone; Ac, acetylation. The data in panel I were obtained from Hummels et al. (24). EF-P was purified from strain DK3828 (*ymfI*), digested on the gel with chymotrypsin, and then resolved with tandem mass spectrometry. MS/MS spectra of chymotrypsin-digested EF-P with pentenone (P) on Lys32 (*m/z* = 407.8966; *z* = 4+). Download FIG S3, PDF file, 0.7 MB. Copyright © 2018 Witzky et al. 2018 Witzky et al. This content is distributed under the terms of the Creative Commons Attribution 4.0 International license.

**TABLE 1:**
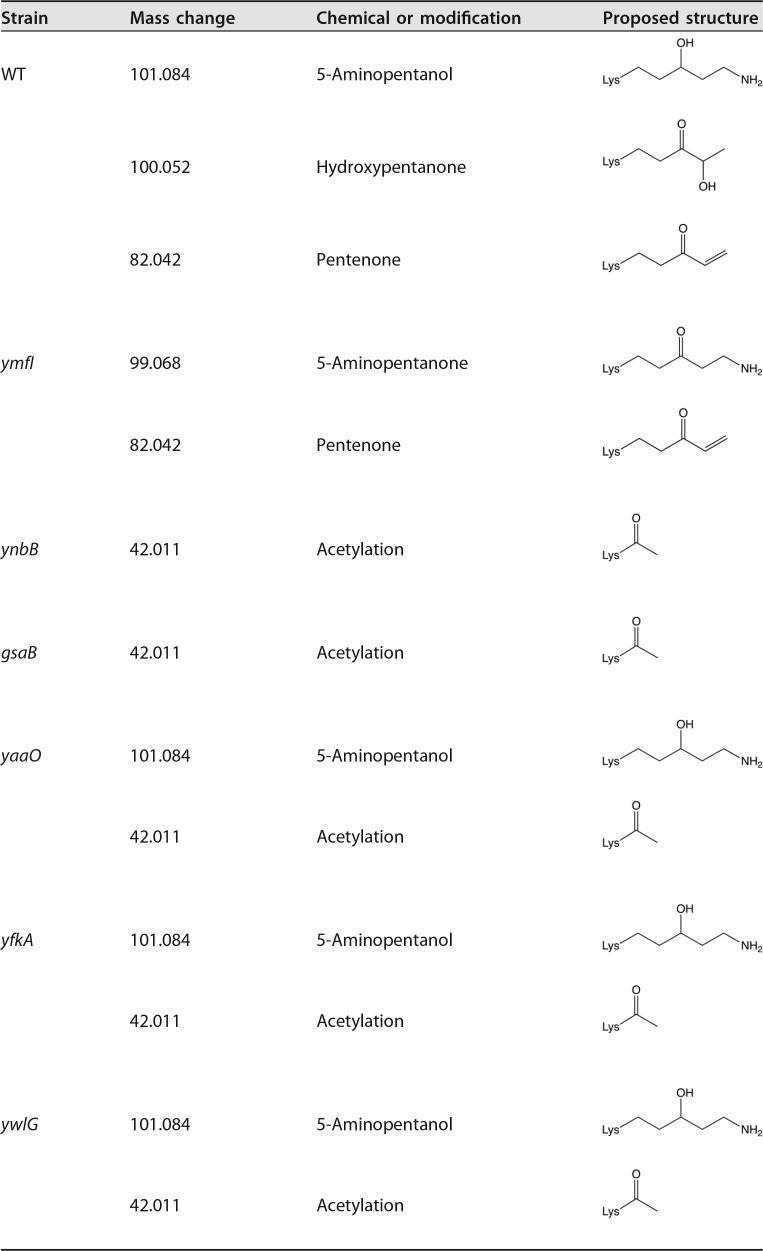
Modifications identified on Lys32

In an effort to assign each enzyme to a step in 5-aminopentanolylation, we searched for all identified intermediates in each mutant data set and in the *ymfI* data set (24). In the *ynbB* and *gsaB* mutants, 5-aminopentanol was not detected on Lys32 (Fig. S3E and S3G). However, in the absence of *ynbB* and *gsaB*, Lys32 was acetylated (Table 1 and Fig. S3F and S3H). Reanalysis of the MS/MS data for the *ymfI* mutant (24) identified pentenone, but not hydroxypentanone or acetylation (Table 1 and Fig. S3I). We propose that deletion of *ymfI* restricts the completion of modification leading to the accumulation of intermediates immediately upstream of 5-aminopentanone. This is consistent with pentenone synthesis immediately preceding 5-aminopentanone reduction in the EF-P modification pathway. In the absence of *yaaO*, *yfkA*, and *ywlG*, Lys32 retained low levels of 5-aminopentanol, indicating that these genes are not strictly required for 5-aminopentanolylation (Table 1 and Fig. S3J and K, S3M and N, S3P and Q). Acetylation could also be identified on Lys32 in each of these mutants (Table 1 and Fig. S3L, S3O, and S3R).

The presence of 5-aminopentanol on Lys32 in the absence of *yaaO*, *yfkA*, and *ywlG* indicates that these genes are not essential for modification. Although the experimental design here did not allow for precise quantitative analysis, it is evident in the extracted ion chromatograms for the unmodified and modified peptides that the level of modification is substantially lower in the absence of each of these genes (Fig. S4). This together with isoelectric focusing data of the native protein (Fig. 2) suggests that while these genes are not essential for modification, they do influence it, potentially through synthesis of the initial precursor. It has previously been observed that low levels of EF-P modification can be achieved through alternate metabolic routes or environmental acquisition in the absence of canonical modification synthesis genes (15). The results here are consistent with *yaaO*, *ywlG*, and *yfkA* playing a role in synthesis of the substrate, rather than direct modification on EF-P itself.

10.1128/mBio.00306-18.4 FIG S4  Extracted ion chromatograms of unmodified (A, C, E, and G) and modified (B, D, F, and H) EF-P peptides containing Lys32. EF-P-Flag was purified from wild-type (DK2448), Δ*yfkA* (DK572), Δ*ywlG* (DK4573), and *ΔyaaO* (DK3908) strains digested on the gel with trypsin, and resolved on an Orbitrap Elite mass spectrometer (A to F) or Orbitrap fusion mass spectrometer (G and H). Trypsin digestion is inhibited by modification at Lys32, resulting in the peptide VVDFQHVKPGK (*m/z* = 314.1809) when Lys32 is unmodified and VVDFQHVKPGKGAAFVR (*m/z* = 489.7852) when Lys32 is modified. Intensity values are relative to the intensity of the unmodified peptide for the given sample. All chromatograms represent the 4+ ion. Download FIG S4, PDF file, 0.1 MB. Copyright © 2018 Witzky et al. 2018 Witzky et al. This content is distributed under the terms of the Creative Commons Attribution 4.0 International license.

In order to identify the initial substrate, we investigated the proposed activities of PTM genes that have clear homologs with known activities. GsaB is a paralog of HemL, a glutamate-1-semialdehyde aminotransferase that synthesizes 5-aminolevulinic acid, and YaaO is predicted to synthesize cadaverine through lysine decarboxylation. Both 5-aminolevulinic acid and cadaverine bear striking resemblance to 5-aminopentanol. However, supplying either of these substrates into the growth media of the *yaaO* and *gsaB* mutants failed to restore modification of EF-P as assessed by isoelectric focusing (data not shown), indicating that these molecules are not the initial precursor in 5-aminopentanol formation.

**FIG 3:**
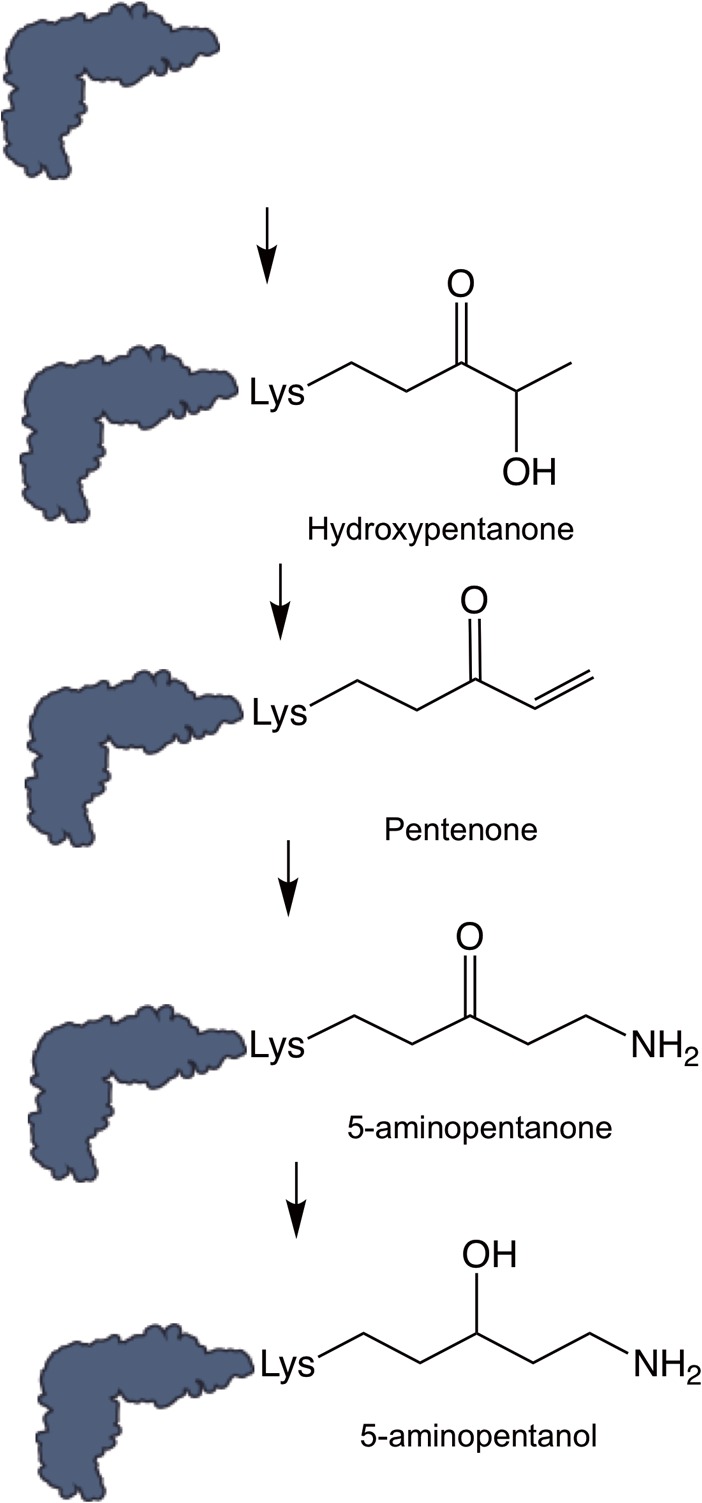
Proposed modification pathway outline based on tandem mass spectrometry analysis. Hummels et al. (24) established that YmfI reduces 5-aminopentanone to 5-aminopentanol in the final step of modification. Additional intermediates identified here represent the most likely structure based on the structure of 5-aminopentanol.

5-Aminopentanol was not detected in the absence of *ynbB*, *gsaB*, and *ymfI*, indicating that these genes are strictly required for modification. YmfI has previously been shown to catalyze reduction of 5-aminopentanone to 5-aminopentanol in the final step of modification (24). As acetylation was the only modification found on Lys32 in the absence of both *ynbB* and *gsaB*, it is difficult to definitively assign each protein to a position in the EF-P PTM pathway. On the basis of sequence similarity to genes of known function, GsaB is predicted to have aminotransferase activity, and YnbB is predicted to have carbon-sulfur lyase activity. On the basis of these proposed activities, it is likely that GsaB facilitates addition of the final amine group onto pentenone and that YnbB removes a fatty acid biosynthesis (FAB) substrate from an acyl carrier protein and forms hydroxypentanone on EF-P, possibly forming the first intermediate in 5-aminopentanol formation. Although an acetyl group was identified in the PTM mutants, the absence of this modification in the wild-type sample suggests that it is not a true intermediate in 5-aminopentanol formation, but rather a spurious side reaction that occurs in the presence of high levels of unmodified EF-P. Taken together, these data suggest a pathway for the 5-aminopentanolylation of EF-P in *B. subtilis* (Fig. 3).

On the basis of the proposed pathway, 5-aminopentanol is assembled directly on EF-P in a series of dehydration/reduction reactions, a type of synthesis that resembles fatty acid biosynthesis. Given these similarities, we hypothesized that 5-aminopentanol is derived from FAB and that assembly could be impacted by changes in this process. To investigate this possibility, EF-P modification state was assessed via isoelectric focusing after knockdown or overexpression of three essential FAB factors (*fabF*, *fabG*, and *accB*) (Fig. S5) (25). Genetic manipulations of these factors did not influence the isoelectric focusing point of EF-P, likely indicating that the FAB and PTM pathway function independently in *B. subtilis*.

10.1128/mBio.00306-18.5 FIG S5  Overexpression (A) or knockdown (B) of fatty acid biosynthesis factors does not influence EF-P modification state. WT, *fabF* overexpression (OE) (AW146), *fabG* OE (AW147), *accB* OE (AW148), *fabF* knockdown (KD) (BEC11340), *fabG* KD (BEC15910), and *accB* KD (BEC24350) strains were grown to mid-log phase in LB or LB supplemented with 1 mM IPTG (A) or 0.1% xylose (B). The lysates were resolved via isoelectric focusing. Purified recombinant EF-P was run as an unmodified control. Blots were probed with polyclonal EF-P antisera. Download FIG S5, PDF file, 0.1 MB. Copyright © 2018 Witzky et al. 2018 Witzky et al. This content is distributed under the terms of the Creative Commons Attribution 4.0 International license.

### Phenotypic characterization of modification mutants.

**FIG 4:**
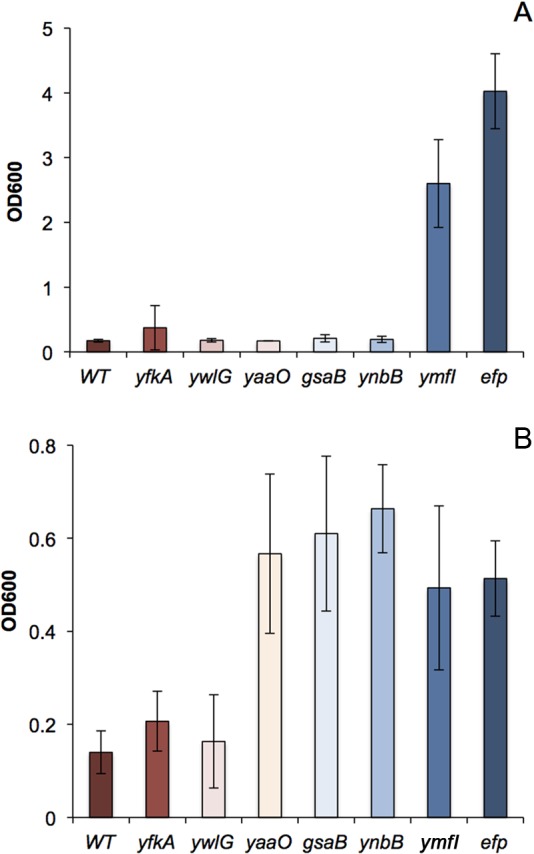
Antibiotic sensitivity in *efp* and PTM mutants. WT (DK1042), *efp* (DK2050), *yfkA* (DK4564), *ywlG* (DK4612), *yaaO* (DK3894), *gsaB* (DK4601), *ynbB* (DK4604), and *ymfI* (DK3621) strains were grown in the presence of the indicated amount of 2 mg/ml sulfanilamide (A) or 15 μg/ml puromycin (B), and the final OD_600_ was measured after 18 h (A) or 8 h (B) of growth.

Given that stable PTM intermediates were identified in each mutant strain, it is possible that EF-P-associated phenotypes are also altered in each of these strains. Consistent with this possibility, swarming motility arrested prematurely in the *gsaB* and *ynbB* mutants (but not in the *yaaO*, *yfkA*, or *ywlG* mutant), and both mutants could be complemented by wild-type alleles (Fig. 1I and Fig. S1C to E). To investigate the impact of the PTM gene deletions in alternative EF-P-associated phenotypes, growth of the WT and *efp* mutant were compared in a Biolog phenotype microarray that tests for 1,920 different metabolic and chemical sensitivities. For the majority of metabolic stressors, the WT and *efp* mutant exhibited no significant growth differences. The WT displayed relatively enhanced respiration in 2,3-butanone (carbon source stress), and the *efp* mutant displayed enhanced respiration in d-glucose-1-phosphate (organic phosphate source stress) (Table S2). Conversely, the *efp* mutant displayed reduced respiration under 60 different chemical stressors with diverse mechanisms of action, indicating that EF-P activity is required under these chemical stressors (Table 2). Many chemicals reduced the growth of the *efp* mutant relative to the wild type, but the *efp* mutant grew better than the wild type in the presence of folate biosynthesis inhibitors like sulfanilamide (Table 3). To determine whether sulfonamide sensitivity is impacted by the EF-P modification state, all modification mutants were grown in the presence of 1 mg/ml to 5 mg/ml sulfanilamide, and growth was measured after 18 h. The majority of modification mutants (*yaaO*, *gsaB*, *ynbB*, *yfkA*, and *ywlG* mutants) did not display the same sulfanilamide resistance seen in the *efp* mutant (Fig. 4A). The exception was the *ymfI* mutant, which was somewhat resistant to sulfanilamide, though not to the full extent of the *efp* mutant (Fig. 4A).

10.1128/mBio.00306-18.7 TABLE S2  Phenotype microarray conditions where WT and Δ*efp* strains display differences in relative growth. Download TABLE S2, PDF file, 0.3 MB. Copyright © 2018 Witzky et al. 2018 Witzky et al. This content is distributed under the terms of the Creative Commons Attribution 4.0 International license.

**TABLE 2:** Growth conditions where the WT displayed enhanced respiration

Mode of action	No. of hits
pH 4.5	19
Toxic ion	14
Chelator	5
Protein synthesis	4
Nitro compound	4
DNA topoisomerase	3
Toxicity	3
Osmotic sensitivity	3
Biofilm inhibitor	3
Cell wall	2
Respiration	2

**TABLE 3:** Growth conditions where the *efp* mutant displayed enhanced respiration

Mode of action	No. of hits
Folate antagonist	9
Oxidizing agent	2
Protein synthesis	1
Cell wall	1

In addition to sulfonamide resistance, the *efp* mutant also displayed enhanced respiration in the presence of puromycin, a translation inhibitor that results in premature termination of polypeptide synthesis (Table 3 and Table S2). To determine whether puromycin sensitivity is impacted by the EF-P modification state, all modification mutants were grown in the presence of 1 μg/ml to 20 μg/ml puromycin, and growth was measured after 8 h. In contrast to the sulfanilamide phenotype, the majority of modification mutants (*yaaO*, *gsaB*, *ynbB*, and *ymfI* mutants) phenocopied the *efp* mutant, while both the *ywlG* and *yfkA* mutants were sensitive to puromycin treatment as in the case of the wild-type strain (Fig. 4B).

### Altered EF-P modification state has variable impact on polyproline translation.

**FIG 5:**
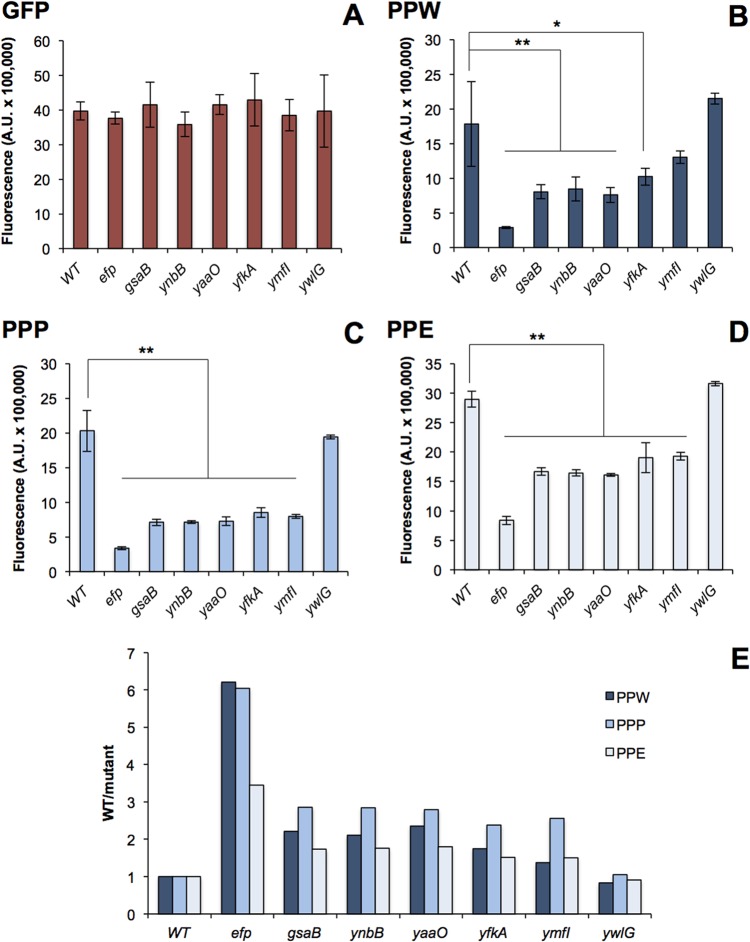
A GFP (A), PPW-GFP (B), PPP-GFP (C), or PPE-GFP (D) reporter construct was chromosomally inserted into *amyE* in each mutant (Rajkovic et al. [22]). After a 1-h induction with 1 mM IPTG, GFP fluorescence was measured. Fluorescence levels were normalized to OD_600_. Values are means ± standard deviations (SD) (error bars) from three biological replicates. Statistical significance was determined with an analysis of variance (ANOVA) and Tukey posthoc test (*, *P* < 0.05; **, *P* < 0.01). (E) Ratio of WT/mutant for each PPX-GFP reporter in each mutant background.

In previously characterized instances of EF-P PTM, EF-P has been shown to require modification for activity, and PTM mutants display phenotypic characteristics similar to those of *efp* mutants (15, 20, 21). Here, phenotypic characterization of the PTM mutants has revealed an apparent discrepancy between the requirement for EF-P itself and the requirement for EF-P modification. One way to explain the discrepancy is if each of the PTM mutants has variation in polyproline translation defects. In order to address this possibility, all mutants were transformed with a polyproline-green fluorescent protein (GFP) reporter construct containing one of three polyproline motifs (PPP, PPW, and PPE) (22). In the absence of *ywlG*, there was no significant defect in polyproline translation for any motif (Fig. 5B to D). In contrast, mutants that lacked *gsaB*, *ynbB*, or *yaaO* displayed significant polyproline translation defects for all three polyproline motifs, although this defect was not as significant as the defect seen in an *efp* mutant (Fig. 5B to D). Loss of *ymfI* or *yfkA* has variable impact on polyproline translation. In the *ymfI* mutant, there is no significant decrease in PPW-GFP levels, whereas there is a significant decrease in PPP-GFP and PPE-GFP levels (Fig. 5B to D). In the *yfkA* mutant, there is a small defect in PPW-GFP levels and more significant decrease in PPP-GFP and PPE-GFP levels. It is also noteworthy that while there is a decrease in polyproline translation efficiency for the *ymfI* and *yfkA* mutants, it is less severe than for *gsaB*, *ynbB*, and *yaaO* mutants.

To characterize the relative severity of the polyproline translational defect for each motif, the average PPX-GFP fluorescence in the wild-type strain was compared to each mutant (Fig. 5E). Consistent with previous studies, PPW-GFP displayed the highest EF-P dependence in the *efp* mutant (5, 22). In contrast, in each of the PTM mutants, PPP-GFP displayed the highest EF-P dependence. Each PTM mutant also displayed variability in the relative EF-P dependence for each motif. Many mutants exhibited a more significant defect in PPW translation than in PPE translation, but in the case of *ymfI* and *ywlG*, the translational defects with both of these motifs were essentially indistinguishable. This indicates that modulation of EF-P modification state not only impacts the strength of EF-P-dependent pausing, but it also alters the context dependence of EF-P-dependent pausing.

## DISCUSSION

### EF-P is modified through multistep assembly that is reminiscent of FAB.

In order to efficiently stimulate translation of polyproline motifs, EF-P requires PTM of a conserved residue. In Gram-negative bacteria and eukaryotes, several diverse modifications have been identified, and the enzymes that facilitate modification are known. Recently, a novel EF-P PTM has been identified in the Gram-positive bacterium *B. subtilis*, but many of the genes required for modification have remained unknown (22). Mass spectrometry analyses revealed that intermediate modifications (5-aminopentanone, hydroxypentanone, pentenone, and acetylation) have been detected on Lys32 in WT and modification-deficient samples. On the basis of the intermediates identified, we propose an outline for EF-P PTM in *B. subtilis* (Fig. 3). It should be noted that intermediates would not have be detected in this study if they were unstable or rapidly turned over. Therefore, additional intermediates in the pathway cannot be excluded. Nevertheless, our results indicate that *B. subtilis* employs a novel multistep method of modification that produces intermediates with distinct similarities to those found in FAB (Fig. 3). Although 5-aminopentanolylation does appear to be derived from FAB, genetic manipulation of FAB did not impact the isoelectric focusing point of EF-P (Fig. S5). This could indicate that the two processes are not biologically linked. It is also likely that the metabolic burden that EF-P modification imposes on the cell is minor compared to that of FAB. Therefore, altering the levels of several FAB factors would not be sufficient to impact the overall EF-P modification substrate pool to the point that changes in EF-P modification status could be readily observed.

Several of the intermediates identified here were not detected in previous studies, possibly because in this study EF-P-FLAG was purified using milder purification conditions to help with the detection of less stable intermediates. Furthermore, in our mass spectrometry procedure, the top 10 most abundant ions from each cycle were selected for MS/MS. In previous studies, only the top four were selected. Together, these differences in methodology allowed for the detection of ions and thus corresponding intermediates that would be less stable or present at much lower abundance.

### 5-Aminopentanolylation as an EF-P modification strategy in other organisms.

**FIG 6:**
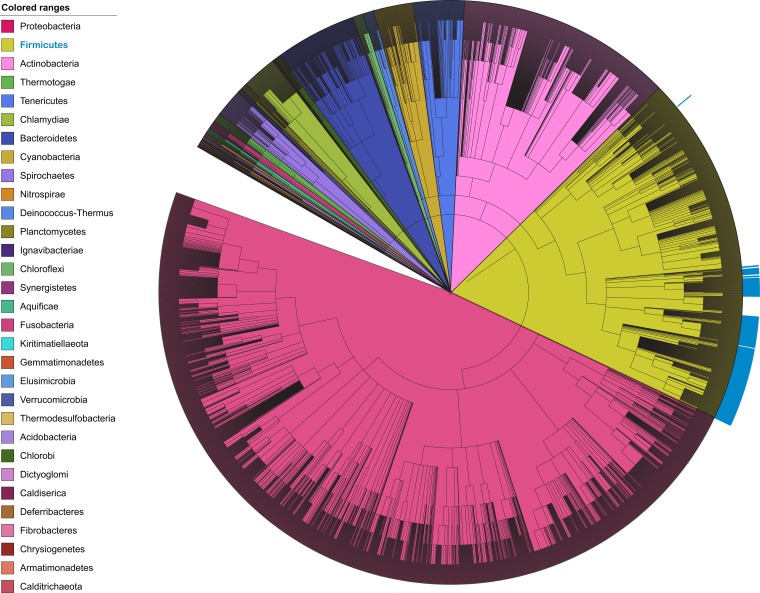
Phylogenetic tree predicting other bacteria that will employ 5-aminopentanolylation as a modification strategy. Organisms that maintain *ymfI*, *ynbB*, *gsaB*, *yfkA*, *ywlG*, and *yaaO* are indicated in blue. Phylogenetic classifications are marked by the corresponding colors in the color key. The taxonomic tree was generated using iTol.

Here, we employed a forward genetic screen to identify genes required for 5-aminopentanolylation of EF-P. In order to predict which other bacteria modify EF-P with 5-aminopentanol, we searched for organisms that maintain all of the *ymfI*, *yaaO*, *gsaB*, *ynbB*, *yfkA*, and *ywlG* genes*.* Although many bacteria contained at least one of the modification genes, organisms that maintain the entire core set of modification genes could be found only within *Firmicutes* (Fig. 6). A number of proteobacteria also appear to maintain the core set of 5-aminopentanolylation genes. As these species also contain the machinery for *R*-β-lysylation, it is unlikely that 5-aminopentanolylation is the EF-P modification strategy for these organisms. This result instead likely stems from the fact that the genes required for 5-aminopentanol assembly are homologous to other broadly conserved genes.

### EF-P-dependent pausing is variably impacted by altered modification state.

In all known instances of EF-P and eIF5A PTM, EF-P requires modification for function, and PTM mutants essentially phenocopy *efp* mutants (15–17, 20). Conversely in *B. subtilis*, there is substantial phenotypic variability between the *efp* mutant and each PTM mutant. The complete 5-aminopentanol PTM is required for puromycin sensitivity, but it is dispensable for swarming motility and sulfanilamide sensitivity in the majority of the PTM mutants. *In vivo* polyproline reporter data indicate that each mutant displays a context-dependent defect in polyproline translation, with each motif being impacted unequally by the altered modification state of EF-P. This result likely accounts for the phenotypic variability observed between the *efp* mutant and the PTM mutants. It is of note that the majority of the modification mutants displayed a polyproline translation defect equal to or more severe than the defect of the *ymfI* mutant, yet they restored swarming to the *ymfI* mutant in the initial screen. This result suggested that PPP, PPE, and PPW are not relevant motifs for swarming proficiency. Of the proteins that are known to be required for motility, 23 contain polyproline motifs, with 13 different PPX motifs distributed among the proteins (22). It is likely that in the absence of *ymfI*, there is a significant polyproline translation defect in one of these other EF-P-dependent proteins, resulting in the swarming defect that can be alleviated by blocking 5-aminopentanone formation.

It has recently been established that in the case of *R*-β-lysylated EF-P, the modification interacts with the CCA end of the p-site tRNA, stabilizing it in a conformation that is favorable for peptide bond formation (11). Here, we have shown that alteration of EF-P modification not only decreases polyproline translation efficiency but also modulates the relative impact that EF-P has on each PPX motif. This result suggests that the relative EF-P dependence of each PPX motif is directly impacted by the nature of the modification itself. It would therefore be expected that organisms with a high content in a specific PPX motif would require a modification suited for a stabilizing conformation for that motif, perhaps justifying the structural diversity found in all known EF-P PTMs. Ribosomal profiling in organisms that employ alternative modification strategies will be required to directly address this possibility.

While EF-P modification is critical for efficient polyproline translation, both the structure and source of the modification are variable between organisms. This speaks to the nature of EF-P posttranslational modification, in that although modifications are structurally diverse, they may all be simply siphoned out of natural metabolic processes. Through this work, we have expanded the possible metabolic sources for EF-P PTM and have shown how alteration of this modification modulates EF-P activity.

## MATERIALS AND METHODS

### Growth conditions and strain construction.

Strains were grown in Luria broth (LB) (5 g NaCl, 5 g yeast extract, 10 g tryptone [all per  liter]) with 100 μg/ml spectinomycin, 12.5 μg/ml tetracycline, 0.5 μg/ml erythromycin, 5 μg/ml chloramphenicol, 5 μg/ml kanamycin, 1 µg/ml erythromycin plus 25 µg/ml lincomycin (for macrolide-lincomycin-streptogramin B [MLS] resistance), and 100 μg/ml ampicillin when appropriate.

### In-frame deletions. (i) Δ*yaaO*.

*Bacillus subtilis* 3610 genomic DNA was PCR amplified using primers 4933/4934 and 4935/4936. The resulting fragments were inserted into the SmaI restriction site of pMiniMAD2 using Gibson assembly to create pKRH58. pKRH58 was passaged through the *recA*^+^ *Escherichia coli* strain TG1 and subsequently transformed into strain DK1042 or DK4601. pMiniMAD2 encodes MLS resistance and a temperature-sensitive origin of replication that is active at room temperature, but not at 37°C. Thus, colonies that integrated the plasmid into their genome were selected for by growth in the presence of MLS at 37°C. Plasmid eviction was promoted by inducing the temperature-sensitive origin through overnight growth at room temperature. The resulting MLS-sensitive colonies were isolated and confirmed to carry the deletion by PCR-length polymorphism analysis to create strains DK3894 (Δ*yaaO*) and DK4750 (Δ*gsaB* Δ*yaaO*). The transposon from strain DK4300 was transduced into DK1042 to produce DK4325.

### (ii) Δ*yfkA*.

*B*. *subtilis* 3610 genomic DNA was PCR amplified using primers 5279/5281 and 5280/5282. The resulting fragments were inserted into the SmaI restriction site of pMiniMAD2 using Gibson assembly to create pKRH69. pKRH69 was passaged through the *recA*^+^ *E. coli* strain TG1 and subsequently transformed into strain DK1042. MLS-resistant colonies were isolated at 37°C, and plasmid eviction was promoted by overnight growth at room temperature. The resulting MLS-sensitive colonies were isolated and confirmed to carry the deletion by PCR-length polymorphism analysis to create strain DK4564. The *ymfI*::*tet* mutation was transduced into strain DK4564 to produce DK5170. The transposon from strain DK4303 was transduced into DK1042 to produce DK4555.

### (iii) Δ*ynbB*.

*B*. *subtilis* 3610 genomic DNA was PCR amplified using primers 5283/5284 and 5285/5286. The resulting fragments were inserted into the SmaI restriction site of pMiniMAD2 using Gibson assembly to create pKRH70. pKRH70 was passaged through the *recA*^+^ *E. coli* strain TG1 and subsequently transformed into strain DK1042. MLS-resistant colonies were isolated at 37°C, and plasmid eviction was promoted by overnight growth at room temperature. The resulting MLS-sensitive colonies were isolated and confirmed to carry the deletion by PCR-length polymorphism analysis to create strain DK4604. The *ymfI*::*tet* mutation was transduced into strain DK4604 to produce DK5172. The transposon from strain DK4381 was transduced into DK1042 to produce DK4389.

### (iv) Δ*ynbA*.

*B*. *subtilis* 3610 genomic DNA was PCR amplified using primers 5287/5288 and 5289/5290. The resulting fragments were inserted into the SmaI restriction site of pMiniMAD2 using Gibson assembly to create pKRH71. pKRH71 was passaged through the *recA*^+^ *E. coli* strain TG1 and subsequently transformed into strain DK1042. MLS-resistant colonies were isolated at 37°C, and plasmid eviction was promoted by overnight growth at room temperature. The resulting MLS-sensitive colonies were isolated and confirmed to carry the deletion by PCR-length polymorphism analysis to create strain DK4605. The *ymfI*::*tet* mutation was transduced into strain DK4605 to produce DK5173.

### (v) Δ*gsaB*.

*B*. *subtilis* 3610 genomic DNA was PCR amplified using primers 5291/5292 and 5293/5294. The resulting fragments were inserted into the SmaI restriction site of pMiniMAD2 using Gibson assembly to create pKRH72. pKRH72 was passaged through the *recA*^+^ *E. coli* strain TG1 and subsequently transformed into strain DK1042. MLS-resistant colonies were isolated at 37°C, and plasmid eviction was promoted by overnight growth at room temperature. The resulting MLS-sensitive colonies were isolated and confirmed to carry the deletion by PCR-length polymorphism analysis to create strain DK4601. The *ymfI*::*tet* mutation was transduced into strain DK4601 to produce DK5171. The transposon from strain DK4375 was transduced into DK1042 to produce DK4554.

### (vi) Δ*ywlG*.

*B*. *subtilis* 3610 genomic DNA was PCR amplified using primers 5295/5296 and 5297/5298. The resulting fragments were inserted into the SmaI restriction site of pMiniMAD2 using Gibson assembly to create pKRH73. pKRH73 was passaged through the *recA*^+^ *Escherichia coli* strain TG1 and subsequently transformed into strain DK1042. MLS-resistant colonies were isolated at 37°C, and plasmid eviction was promoted by overnight growth at room temperature. The resulting MLS-sensitive colonies were isolated and confirmed to carry the deletion by PCR-length polymorphism analysis to create strain DK4612. The *ymfI*::*tet* mutation was transduced into strain DK4601 to produce DK5174. The transposon from strain DK4368 was transduced into DK1042 to produce DK4556.

### EF-P-FLAG expression strains.

To create the *yaaO efp-flag* strain, the *efp*::*tet* mutation from strain DS354 was transduced into strain DK3894, and the *amyE*::*P_hyspank_-efp-flag* construct from strain DK2448 was transduced into the resulting strain to create DK3908. To create the *yfkA efp-flag* strain, the *efp*::*tet* mutation from strain DS354 was transduced into DK4564, and the *amyE*::*P_hyspank_-efp-flag* construct from DK2448 was transduced into the resulting strain to create DK4572. To create the *gsaB yaaO efp-flag* strain, the *efp*::*tet* mutation from strain DS354 was transduced into DK4750, and the *amyE*::*P_hyspank_-efp-flag* construct from DK2448 was transduced into the resulting strain to create DK4815. The *ynbB*, *gsaB*, and *ywlG efp-flag* strains were created by transducing the transposons from strains DK4301, DK4304, and DK4368 into strain DK2448 to create DK4310, DK4313, and DK4573, respectively.

### Complementation constructs. (i) *P*_*ynbA*_-*ynbB*.

The *ynbA* promoter was amplified using primers 5877/5878, and the *ynbB* open reading frame was amplified using primers 5879/5880 from *B*. *subtilis* 3610 genomic DNA. The *ynbA* and *ynbB* fragments were digested with HindIII/NheI and NheI/SphI, respectively, and ligated into the HindIII/SphI sites of pAH25 to create pKRH86. pKRH86 was transformed into strain DK4604 to create DK5321, and the *ymfI*::*tet* mutation from strain DS235 was transduced into DK5309 to create DK5321.

### (ii) *P*_*yfkA*_-*yfkA*.

The *yfkA* promoter and open reading frame were amplified using primers 5881/5882 from *B*. *subtilis* 3610 genomic DNA. The resulting fragment was digested with BamHI/NheI and ligated into the BamHI/NheI sites of pAH25 to create pKRH87. pKRH87 was transformed into strain DK4564 to create DK5165, and the *ymfI*::*tet* mutation from strain DS235 was transduced into DK5165 to create DK5304.

### (iii) *P*_*ynbA*_-*ynbB*.

The *ywlF* promoter was amplified using primers 5883/5884, and the *ywlG* open reading frame was amplified using primers 5879/5880 from *B*. *subtilis* 3610 genomic DNA. The *ywlF* and *ywlG* fragments were digested with HindIII/NheI and NheI/EcoRI, respectively, and ligated into the HindIII/EcoRI sites of pAH25 to create pKRH88. pKRH88 was transformed into strain DK4612 to create DK5166, and the *ymfI*::*tet* mutation from strain DS235 was transduced into DK5166 to create DK5326.

### (iv) ***P*****_*xpcA*_-*yaaO***.

The *xpcA* promoter was amplified using primers 5887/5888, and the *yaaO* open reading frame was amplified using primers 5889/5890 from *B*. *subtilis* 3610 genomic DNA. The *xpcA* and *yaaO* fragments were digested with HindIII/NheI and NheI/SphI, respectively, and ligated into the HindIII/SphI sites of pAH25 to create pKRH89. pKRH89 was transformed into strain DK3894 to create DK5167, and the *ymfI*::*tet* mutation from strain DS235 was transduced into DK5167 to create DK5305.

### (v) *P*_*gsaB*_-*gsaB*.

The *gsaB* promoter and open reading frame were amplified using primers 5909/5946 from *B*. *subtilis* 3610 genomic DNA. The resulting fragment was digested with BamHI/NheI and ligated into the BamHI/NheI sites of pAH25 to create pKRH90. pKRH90 was transformed into strain DK4601 to create DK5308, and the *ymfI*::*tet* mutation from strain DS235 was transduced into DK5308 to create DK5320.

### (vi) *P*_*hyspank*_-*yaaO.*

The *yaaO* open reading frame was excised from pKRH89 by restriction digestion with NheI/SphI and ligated into the NheI/SphI restriction sites of pDR111 to create pKRH112. pKRH112 was then transformed into strains DK3894 and DK4077 to create DK5327 and DK5328, respectively.

### PPX-GFP reporter strains. (i) *amyE*::*P*_*hyspank*_-*gfp*.

pAW92 was transformed into strains DK1042, DK2050, DK4601, DK4604, DK4564, DK4612, DK3621, and DK3894 to create strains AW112, AW114, AW118, AW120, AW122, AW124, RT01, and RT03, respectively (22).

### (ii) *amyE*::*P*_*hyspank*_-*ppw*-*gfp*.

pAW40 was transformed into strains DK1042, DK2050, DK4601, DK4604, DK4564, DK4612, DK3621, and DK3894 to create strains AW113, AW115, AW119, AW121, AW123, AW125, RT02, and RT04, respectively (22).

### (iii) *amyE*::*P*_*hyspank*_-*ppp*-*gfp*.

pAW93 was transformed into strains DK1042, DK2050, DK3621, DK3894, DK4601, DK4604, DK4564, and DK4612 to create strains AW149, AW150, AW156, AW157, AW158, AW159, AW160, and AW161, respectively (22).

### (iv) *amyE*::*P*_*hyspank*_-*ppe*-*gfp*.

*ppe-gfp* was amplified from pAW93 using primers 1657/7514. The resulting fragment was ligated into SphI/NheI-digested pDR111 using Gibson assembly to generate pAW162. pAW162 was transformed into strains DK1042, DK2050, DK3621, DK3894, DK4601, DK4604, DK4564, and DK4612 to create strains AW163, AW164, AW165, AW166, AW167, AW168, AW169, and AW170, respectively.

### FAB overexpression strains. (i) *amyE*::*P_hyspank_-fabF*.

*fabF* was amplified from *B. subtilis* 3610 genomic DNA using primers 6102/6103. The resulting fragment was ligated into SphI/NheI-digested pDR111 using Gibson assembly to generate pAW143. pAW143 was transformed into strain DK1042 to generate strain AW146.

### (ii) *amyE*::*P*_*hyspank*_-*fabG*.

*fabG* was amplified from *B. subtilis* 3610 genomic DNA using primers 6104/6105. The resulting fragment was ligated into SphI/NheI-digested pDR111 using Gibson assembly to generate pAW144. pAW144 was transformed into strain DK1042 to generate strain AW147.

### (iii) *amyE*::*P*_*hyspank*_-*fabG*.

*fabG* was amplified from *B. subtilis* 3610 genomic DNA using primers 6106/6107. The resulting fragment was ligated into SphI/NheI-digested pDR111 using Gibson assembly to generate pAW145. pAW145 was transformed into strain DK1042 to generate strain AW148.

### YmfI suppressor screen.

Transposon delivery vectors for TnYLB and TnHyJump were introduced into the nonswarming, sensitized *ymfI* mutant background DK3789 by SPP1-mediated phage transduction followed by selection for MLS resistance at room temperature. Both delivery vectors contain a transposon encoding a kanamycin resistance cassette and a temperature-sensitive origin that allows for replication at room temperature but not 42°C in *B. subtilis* (26, 27). The resulting colonies were used to inoculate 26 separate 3-ml LB cultures, and transposon mutagenesis was allowed to occur by incubation at room temperature overnight. Mutants with transposon insertions in the genome were selected by incubating cells at 42°C on LB plates containing kanamycin. Approximately 10,000 of the resulting colonies from each pool were combined, and each pool was used to inoculate separate swarming motility agar plates. Following a 7- to 10-h incubation at 37°C, swarming proficient mutants emerged from the site of inoculation as a disk of motile cells, colonies were isolated, and the transposon was backcrossed to verify that suppression of *ymfI* was inseparably linked to the transposon insertion.

To determine the locations of the transposon insertion sites, genomic DNA was isolated from each backcrossed suppressor strain, digested with either Sau3A1 or TaqA1 restriction enzymes, and ligated using T4 ligase to create circular fragments. Primer pairs 695/696 and 2567/2818, which anneal to TnYLB and TnHyJump, respectively, and direct polymerization outwards from the transposon was used to PCR amplify the neighboring DNA. The resulting DNA fragments were subsequently sequenced with primers 696 and 2567, respectively, to determine the transposon insertion site.

### Swarming motility assay.

Cells were grown to mid-logarithmic phase in LB at 37°C and concentrated to an optical density at 600 nm (OD_600_) of 10 in phosphate-buffered saline (PBS) (pH 7.4) (0.8% NaCl, 0.02% KCl, 100 mM Na_2_HPO_4_, and 17.5 mM KH_2_PO_4_) plus 0.5% India ink. Cell suspensions were used to centrally inoculate 0.7% agar LB plates that had been dried for 10 min open-faced in a laminar flow hood. Swarm plates were dried an additional 12 min after inoculation. The plates were incubated at 37°C. and swarm radius was monitored along the same axis every 30 min for 5.5 h.

### Isoelectric focusing.

Strains were grown in 5 ml LB at 37°C with shaking. When the cultures had reached mid-log phase, the cells were collected and lysed in 25 μl lysis buffer (10% glycerol, 25 mM Tris [pH 7.4], 100 mM NaCl, cOmplete mini EDTA-free protease inhibitor tablet [Roche], 1 mg/ml lysozyme, 1.5 U of DNase I). Isoelectric focusing gels were prepared as previously described, with an ampholyte range of 4.0 to 6.5 (Rajkovic et al. [22]). Prior to sample loading, the isoelectric focusing gel was prefocused at 100 V for 45 min. Following sample loading, the bands were resolved at 200 V for 1 h, 300 V for 1 h, and 500 V for 30 min. The gels were soaked in Towbin buffer for 15 min and then transferred to nitrocellulose paper for Western blotting. Blotting was completed with a 1:40,000 dilution of anti-EF-P polyclonal antisera primary antibody and 1:5,000 dilution of goat anti-rabbit conjugated to horseradish peroxidase secondary antibody. Blots were developed with Bio-Rad Clarity ECL substrate.

### Seminative gel electrophoresis.

Strains were grown to mid-log phase, concentrated to an OD_600_ of 10 in lysis buffer (17.2 mM Tris [pH 7.0], 8.6 mM EDTA [pH 8.0], 1 mg/ml lysozyme, 0.1 mg/ml RNase A, 20 µg/ml DNase I, and 50 µg/ml phenylmethane sulfonyl fluoride) and incubated at 37°C for 30 min. SDS sample buffer (500 mM Tris [pH 6.8], 22% glycerol, 10% SDS, and 0.12% bromophenol blue) was added, and samples were boiled for 5 min. The 12-µl boiled samples were loaded onto 10% polyacrylamide native (with no added SDS) or 15% polyacrylamide denaturing (with 0.1% SDS) gels. The lysates were resolved at 150 V for 1.25 h, transferred onto nitrocellulose membranes, and subsequently probed with a 1:40,000 dilution of anti-EF-P or a 1:80,000 dilution anti-SigA polyclonal antiserum. Following incubation with the primary antibodies, nitrocellulose membranes were probed with horseradish peroxidase-conjugated goat anti-rabbit immunoglobulin G. Blots were developed using Pierce ECL substrate (Thermo Fisher Scientific).

### Mass spectrometry.

EF-P-Flag was purified from strains DK2448, DK4313, DK4310, DK4572, DK4573, and DK3908. Saturated overnight cultures were back diluted 1:1,000 in 1.5 liters of LB. When cultures reached mid-log phase, EF-P-Flag expression was induced with 1 mM IPTG for 3 h. The cells were then collected and lysed in 5 ml lysis buffer (50 mM Tris [pH 7.4], 150 mM NaCl, 1 mg/ml lysozyme, cOmplete mini EDTA-free protease inhibitor tablet [Roche]) for 1 h at 37°C. EF-P-Flag was purified with anti-flag M2 magnetic beads (Sigma-Aldrich) at 4°C, following the manufacturer’s instructions with minor alterations. EF-P was eluted with 100 μg/ml flag peptide (Sigma-Aldrich), concentrated, and resolved on a 13% SDS-polyacrylamide gel. Bands were visualized with colloidal Coomassie blue stain and excised for in-gel digestion with trypsin.

For strains DK2448, DK4313, DK4310, DK4572, and DK4573, the generated peptide samples were brought up in 2% acetonitrile in 0.1% formic acid (20 μl) and analyzed (2 μl) by liquid chromatography-electrospray ionization-tandem mass spectrometry (LC/ESI MS/MS) with a Thermo Scientific Easy-nLC II system (Thermo Fisher Scientific, Waltham, MA) coupled to a hybrid Orbitrap Elite ETD (Thermo Fisher Scientific, Waltham, MA) mass spectrometer. In-line desalting was accomplished using a reversed-phase trap column (100 μm by 20 mm) packed with Magic C_18_AQ (5-μm 200-Å resin; Michrom Bioresources, Auburn, CA) followed by peptide separations on a reversed-phase column (75 μm by 250 mm) packed with Magic C_18_AQ (5-μm 100-Å resin; Michrom Bioresources, Auburn, CA) directly mounted on the electrospray ion source. A 40-min gradient from 2% to 40% acetonitrile in 0.1% formic acid at a flow rate of 400 nl/min was used for chromatographic separations. A spray voltage of 2,750 V was applied to the electrospray tip, and the Orbitrap Elite instrument was operated in the data-dependent mode, switching automatically between MS survey scans in the Orbitrap (AGC target value, 1,000,000; resolution, 120,000; injection time, 250 ms) with MS/MS spectra detected in the Orbitrap (AGC target value, 50,000; resolution, 15,000; injection time, 250 ms). The 10 most intense ions from the Fourier transform (FT) full scan were selected for fragmentation in the Orbitrap using electron transfer dissociation (ETD) 100-ms activation time with supplemental collision-induced dissociation (CID) activation with normalized collision energy of 35%. Selected ions were dynamically excluded for 10 s.

For strains DK2448 and DK3908, the generated peptide samples were brought up in 2% acetonitrile in 0.1% formic acid (20 μl) and analyzed (2 μl) by LC/ESI MS/MS with a Thermo Scientific Easy-nLC II (Thermo Fisher Scientific, Waltham, MA) coupled to a hybrid Orbitrap Fusion (Thermo Fisher Scientific, Waltham, MA) mass spectrometer. The peptides were separated on a reversed-phase column (75 μm by 370 mm) packed with Magic C_18_AQ (5-μm 100-Å resin; Michrom Bioresources, Auburn, CA) directly mounted on the electrospray ion source. A 40-min gradient from 2% to 40% acetonitrile in 0.1% formic acid at a flow rate of 300 nl/min was used for chromatographic separations. A spray voltage of 2,100 V was applied to the electrospray tip, and the Orbitrap Fusion instrument was operated in the data-dependent mode, switching automatically between MS survey scans in the Orbitrap (AGC target value, 400,000; resolution, 120,000; injection time, 50 ms) with MS/MS spectra detected in the Orbitrap (AGC target value, 50,000; resolution, 15,000 resolution; injection time, 22 ms). The 10 most intense ions from the Fourier transform (FT) full scan were selected for fragmentation in the Orbitrap using ETD charge-dependent activation time with CID activation with normalized collision energy of 35%. Selected ions were dynamically excluded for 10 s. Modifications detected when strain DK2448 was analyzed on the Orbitrap Fusion were the same as modifications detected when analyzed on the Orbitrap Elite ETD. MS/MS data are presented only for strain DK2448 analyzed on the Orbitrap Elite ETD, though both data sets are available upon request (Fig. S3J to L). For all samples, peptides were mapped using Proteome Discoverer (Thermo Fisher Scientific).

### GFP reporter assay.

Saturated overnight cultures were back diluted 1:1,000 in LB with 100 μg/ml spectinomycin and grown at 37°C with shaking. When cultures reached mid-log phase, GFP expression was induced with 1 mM IPTG for 1 h. Following induction, 1 ml of cells was collected from each culture and washed once in PBS. Fluorescence was then measured with a Horiba Fluorlog spectrofluorimeter.

### Phenotype microarray.

Respiration of the wild-type (WT) (DK1042) and *efp* mutant (DK2050) strains were compared in the Biolog microbial plates PM1-20 according to the manufacturer’s instructions. Both strains were grown on BUG+B agar plates overnight and then subcultured a second time. Cells were collected from plates and suspended in IF-0a Biolog media to an optical density (OD) of 0.09. Cells were mixed with inoculating fluid additives where appropriate and aliquoted into wells. The plates were incubated in the OmniLog PM System at 37°C for 24 h, with readings taken every hour.

### Antibiotic sensitivity assay.

Overnight saturated cultures were back diluted to an OD_600_ of 0.01 in LB with 0 to 5 mg/ml sulfanilamide or 0 to 20 μg/ml puromycin. The cultures were grown at 37°C with shaking for 18 h or 8 h, respectively. The optical density of each culture was measured after the indicated length of time.

### Phylogenetic analysis.

From the GenBank assembly summary file, we obtained faa files of all bacteria known to have their complete genomes sequenced. The removal of duplicate entries yielded a total of 4,196 genomes from which we built a BLAST database using the NCBI-BLAST-2.3.0+ software (28). The *B. subtilis* 168 genes *efp*, *yaaO*, *ymfI*, *ynbB*, *gsaB*, *yfkA*, and *ywlG* were independently subjected to a BLAST search against the database with an arbitrary E value of 0.001 selected to be the cutoff. The exclusive presence of *efp* (with a lysine at position 32 or analogous to position 32), *yaaO*, *ymfI*, and *ynbB* were plotted across a taxonomic tree generated using ITOL (29). Access to the taxonomic tree can be found at the following web address: http://itol.embl.de/tree/13023848185272891498208300.

10.1128/mBio.00306-18.8 TABLE S3  Strains used in this study. Download TABLE S3, PDF file, 0.5 MB. Copyright © 2018 Witzky et al. 2018 Witzky et al. This content is distributed under the terms of the Creative Commons Attribution 4.0 International license.

10.1128/mBio.00306-18.9 TABLE S4  Plasmids used in this study. Download TABLE S4, PDF file, 0.1 MB. Copyright © 2018 Witzky et al. 2018 Witzky et al. This content is distributed under the terms of the Creative Commons Attribution 4.0 International license.

10.1128/mBio.00306-18.10 TABLE S5  Primers used in this study. Download TABLE S5, PDF file, 0.3 MB. Copyright © 2018 Witzky et al. 2018 Witzky et al. This content is distributed under the terms of the Creative Commons Attribution 4.0 International license.
