# Supplementary figures and images for: EF-P Posttranslational Modification Has Variable Impact on Polyproline Translation in Bacillus subtilis
Source: mBio. 2018 Apr 3;9(2):e00306-18. doi: 10.1128/mBio.00306-18 (PMC5885033; doi:10.1128/mBio.00306-18)

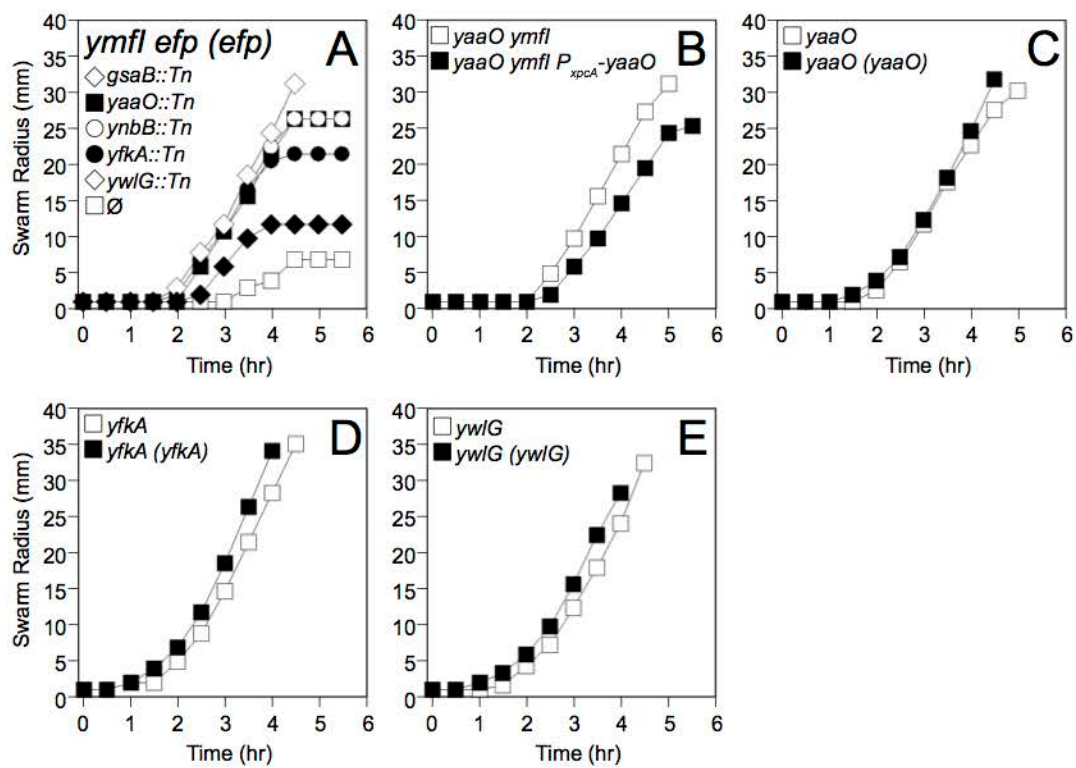

Supplement: FIG S1 [file mbo002183798sf1.pdf]

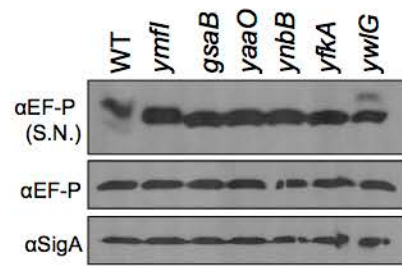

Supplement: FIG S2 [file mbo002183798sf2.pdf]

**Figure S3**

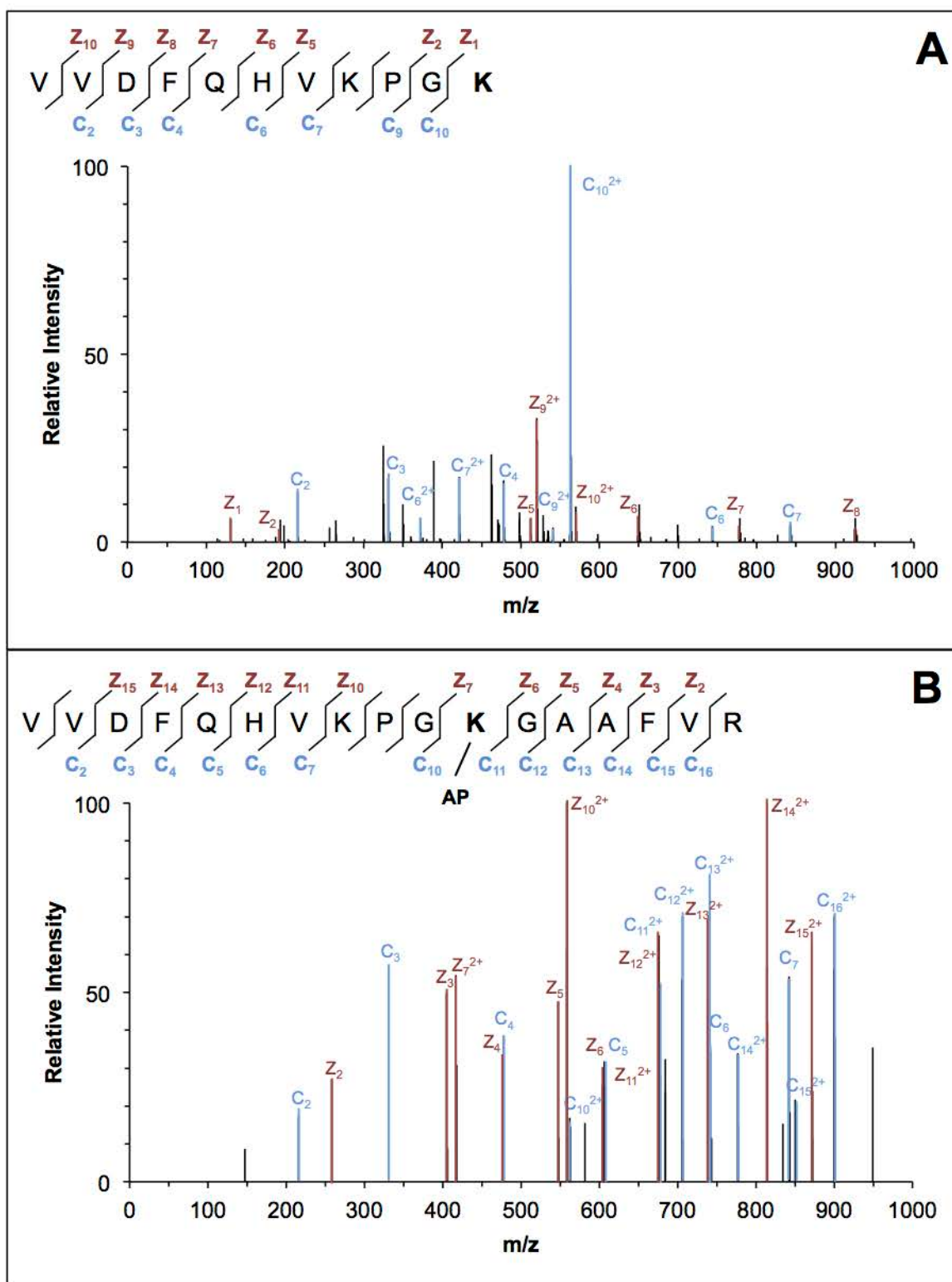

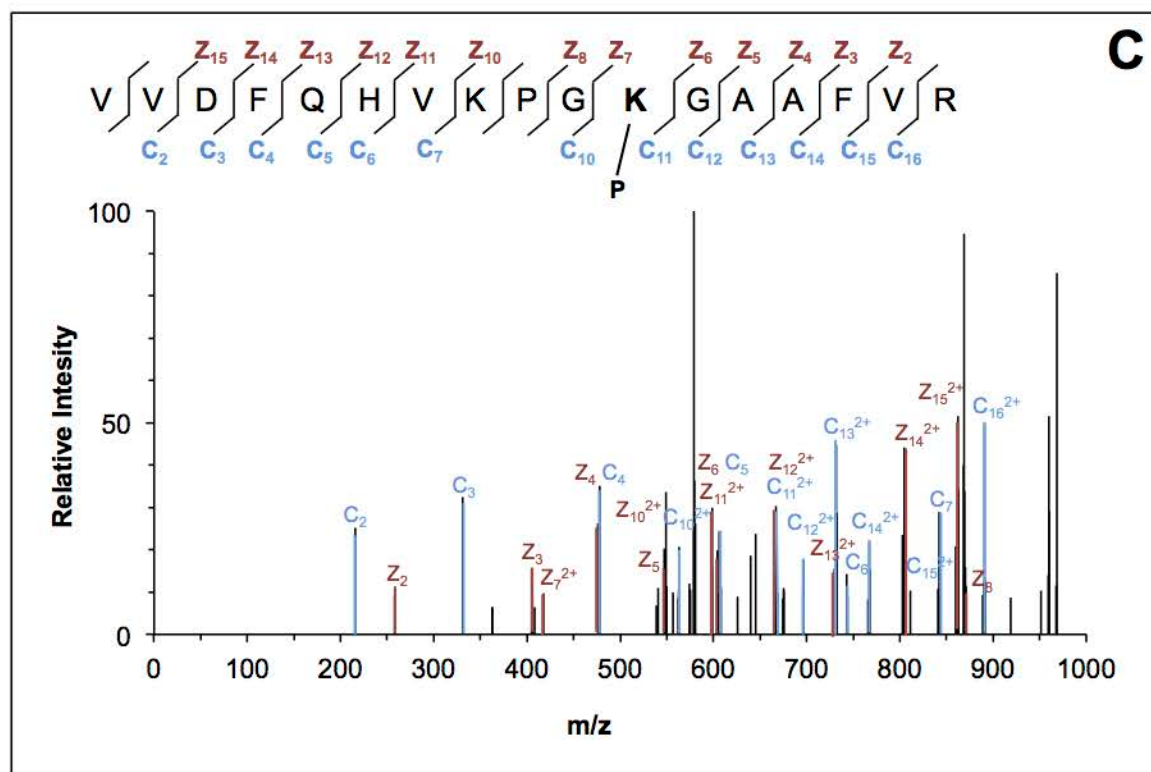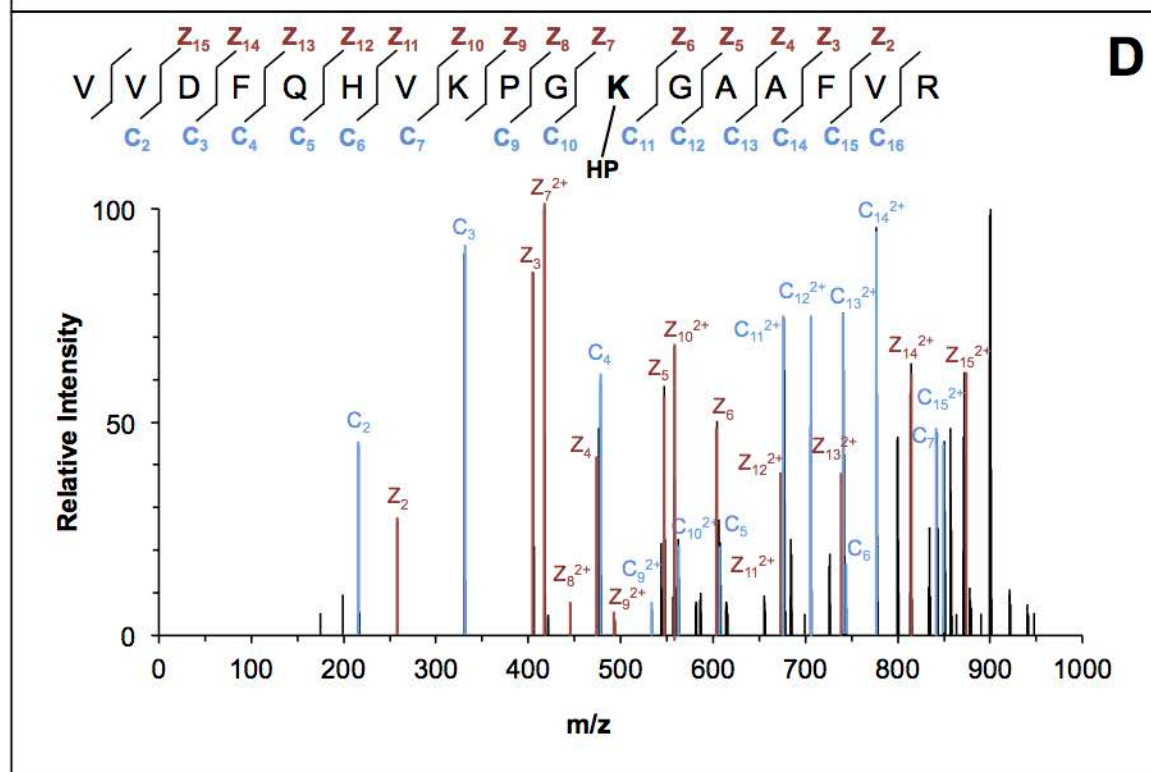

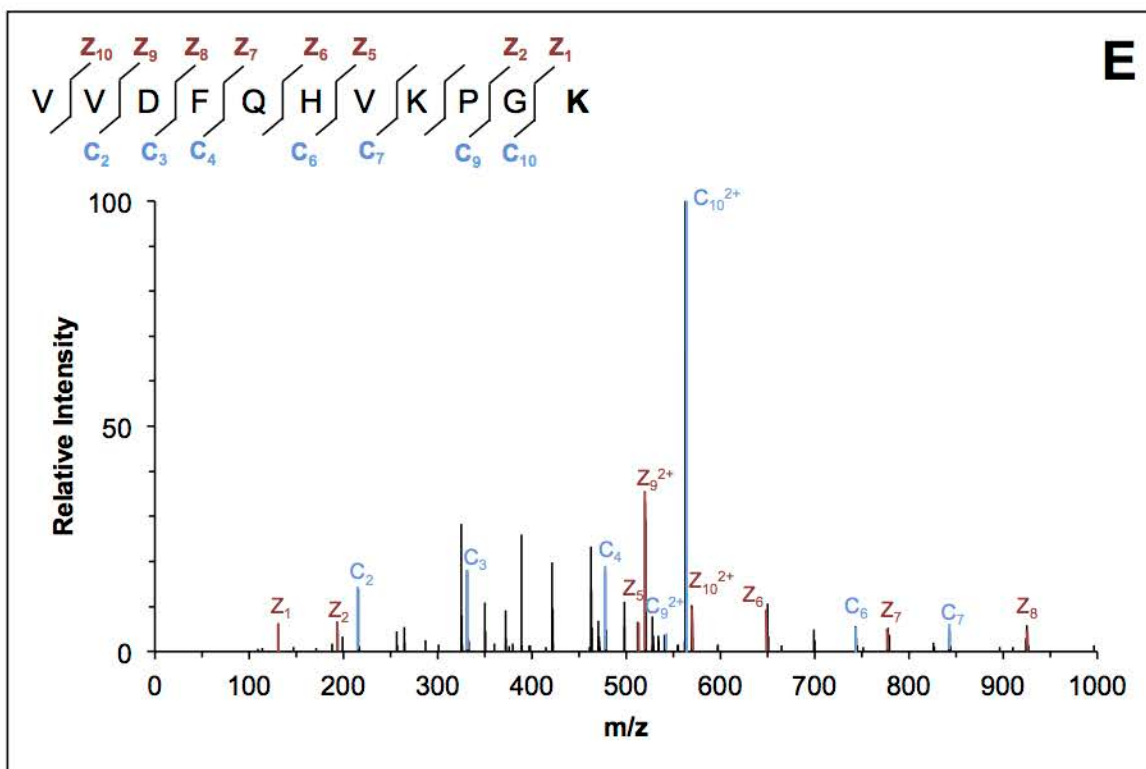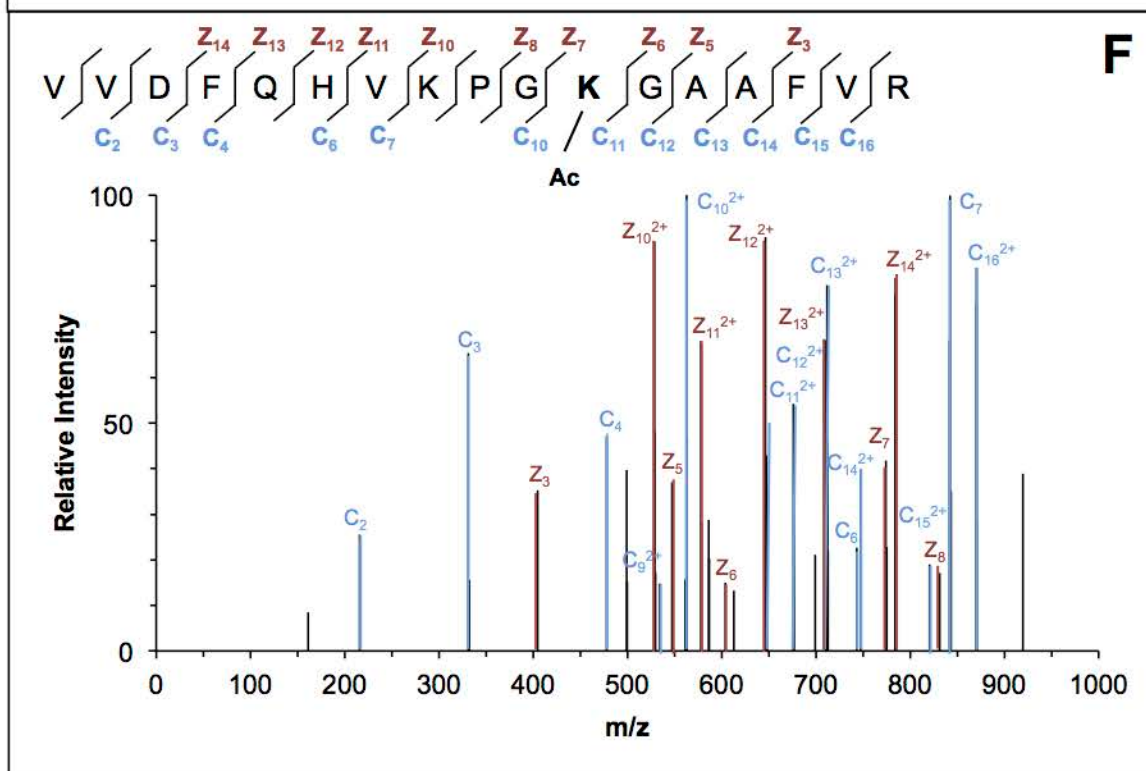

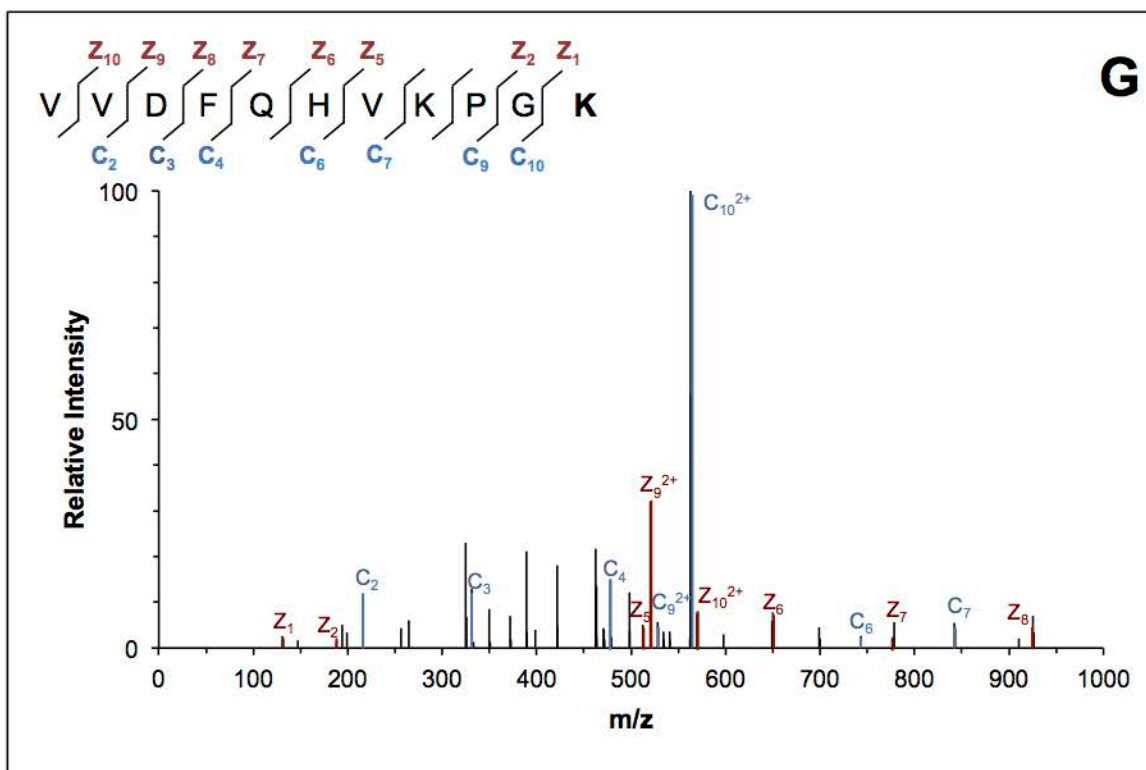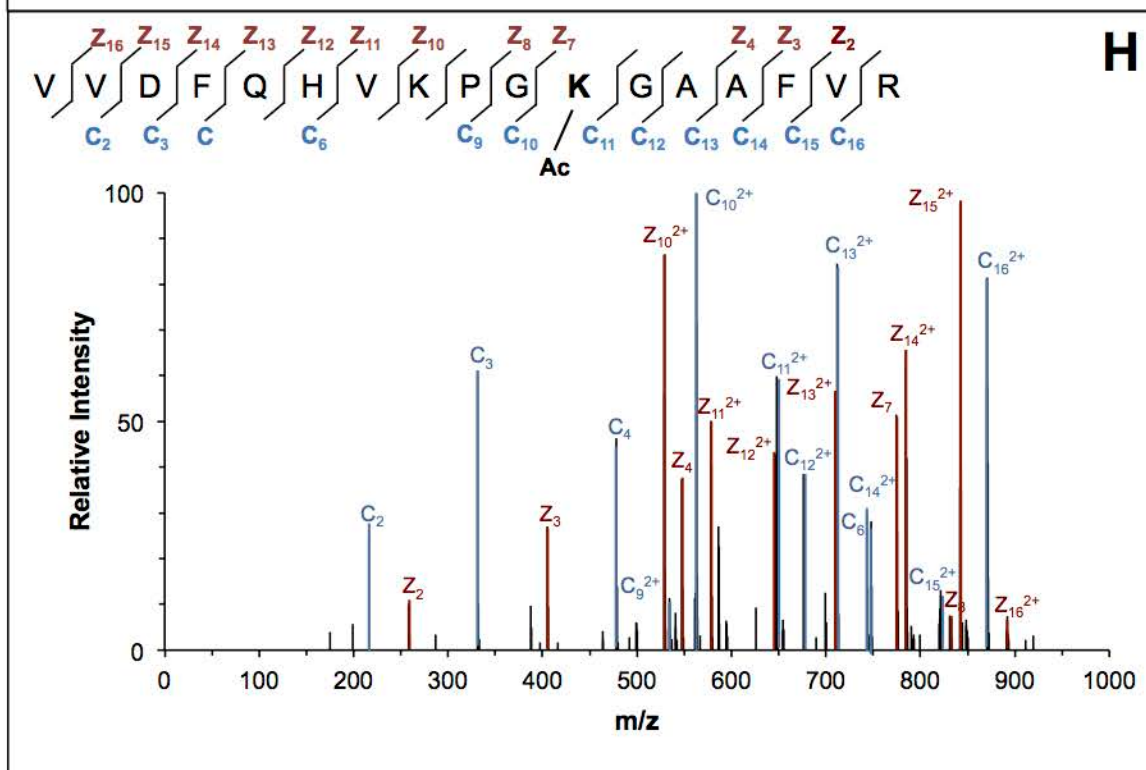

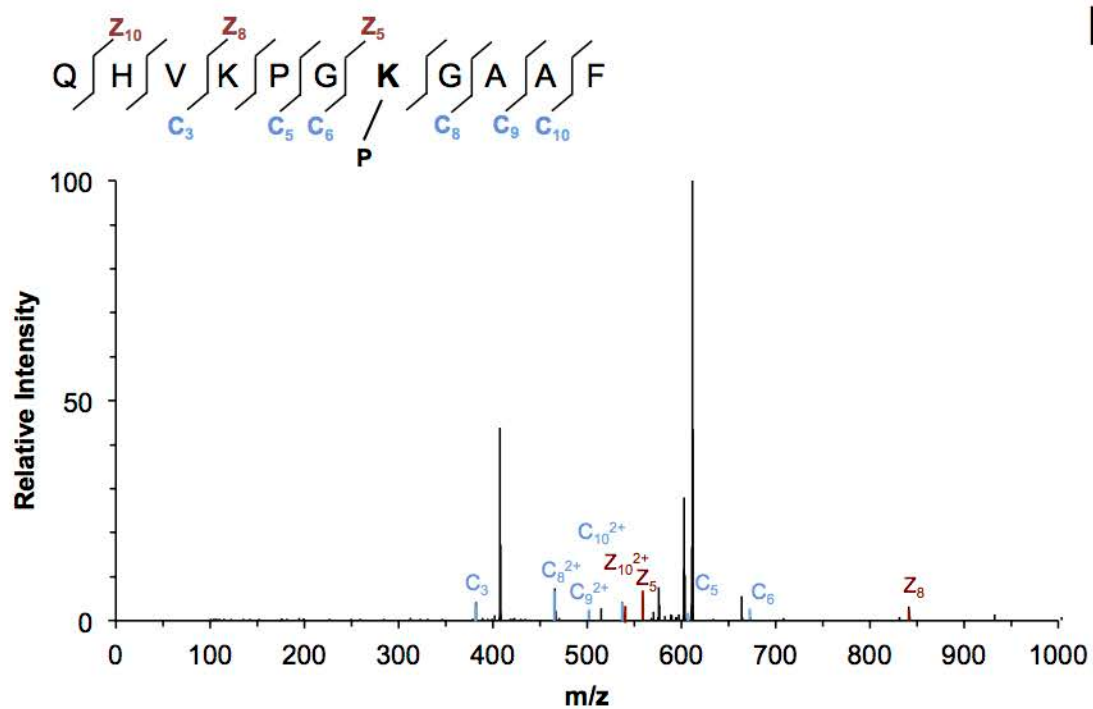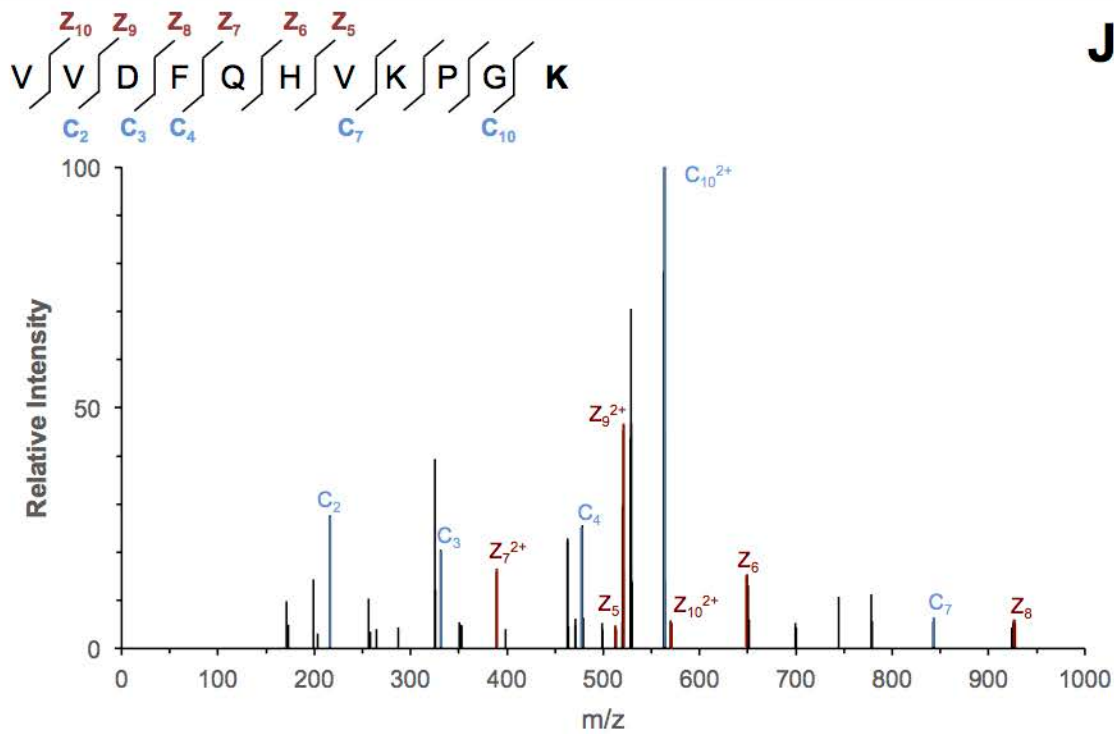

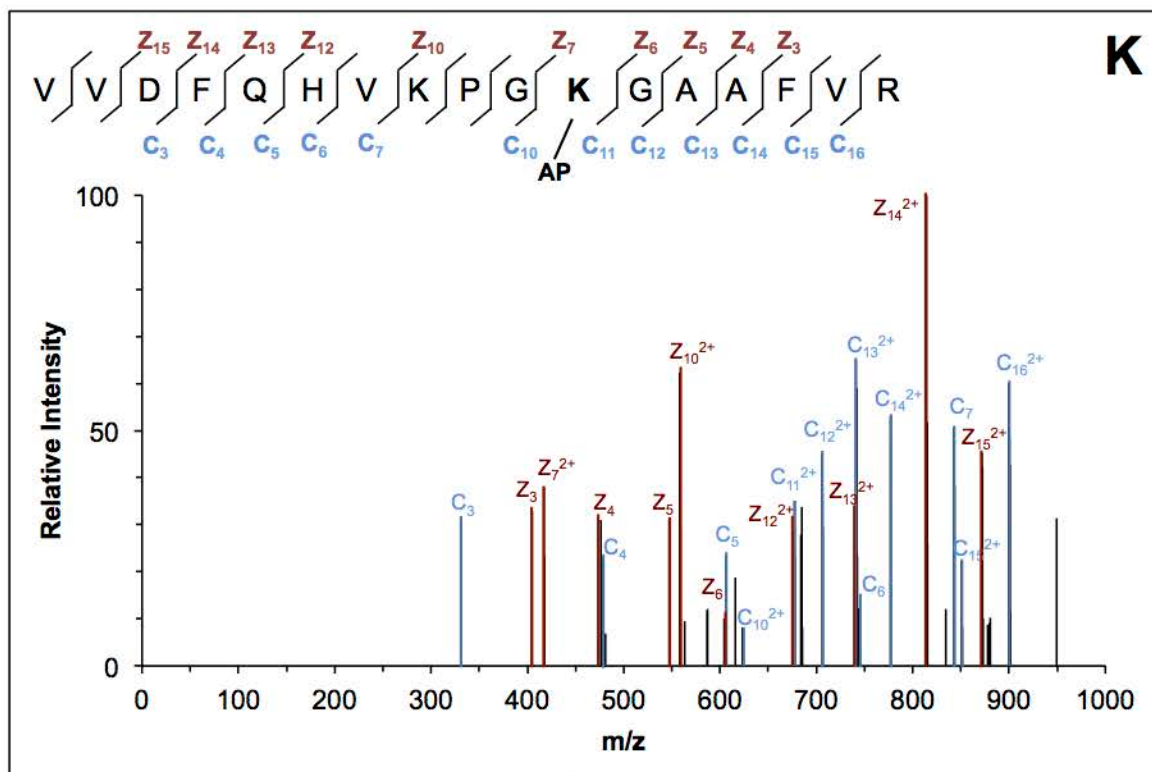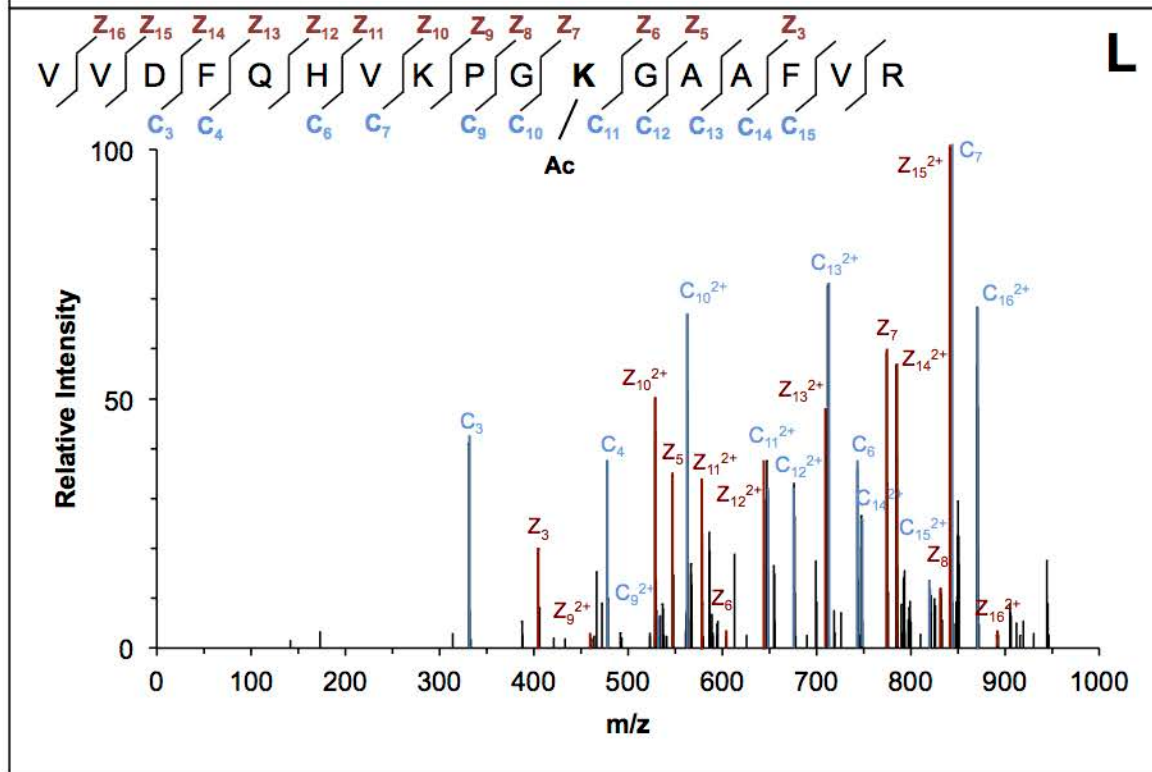

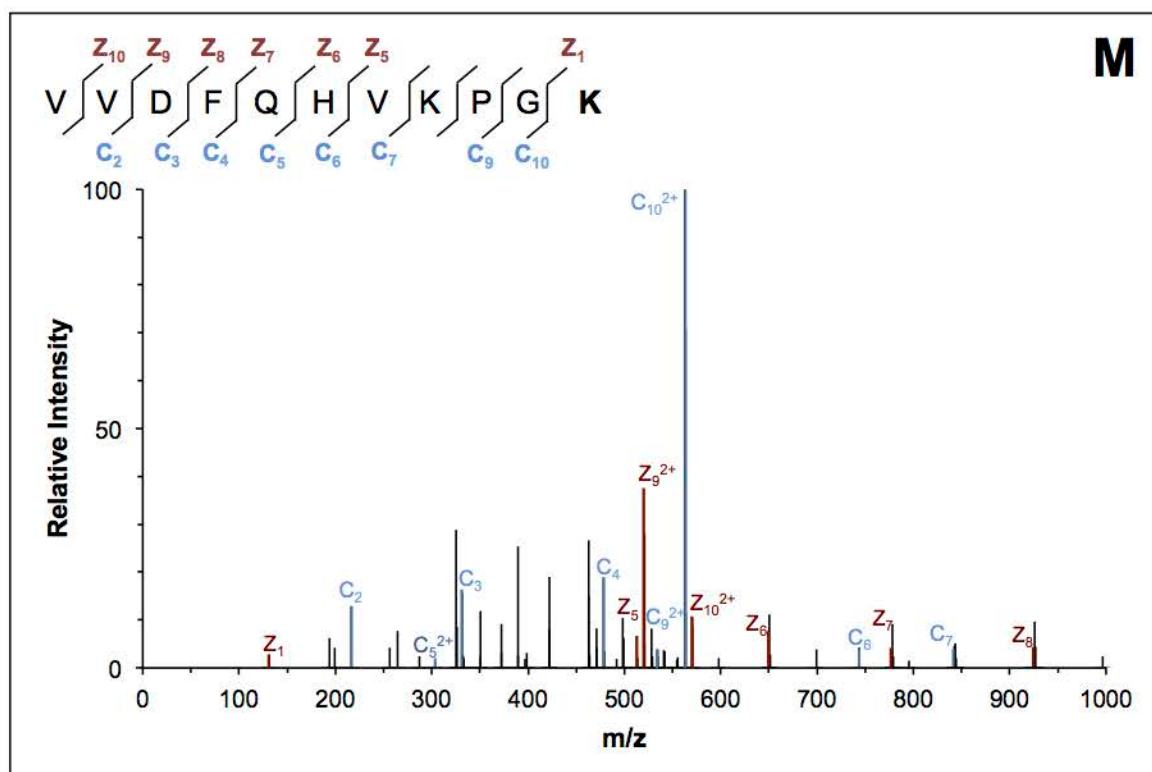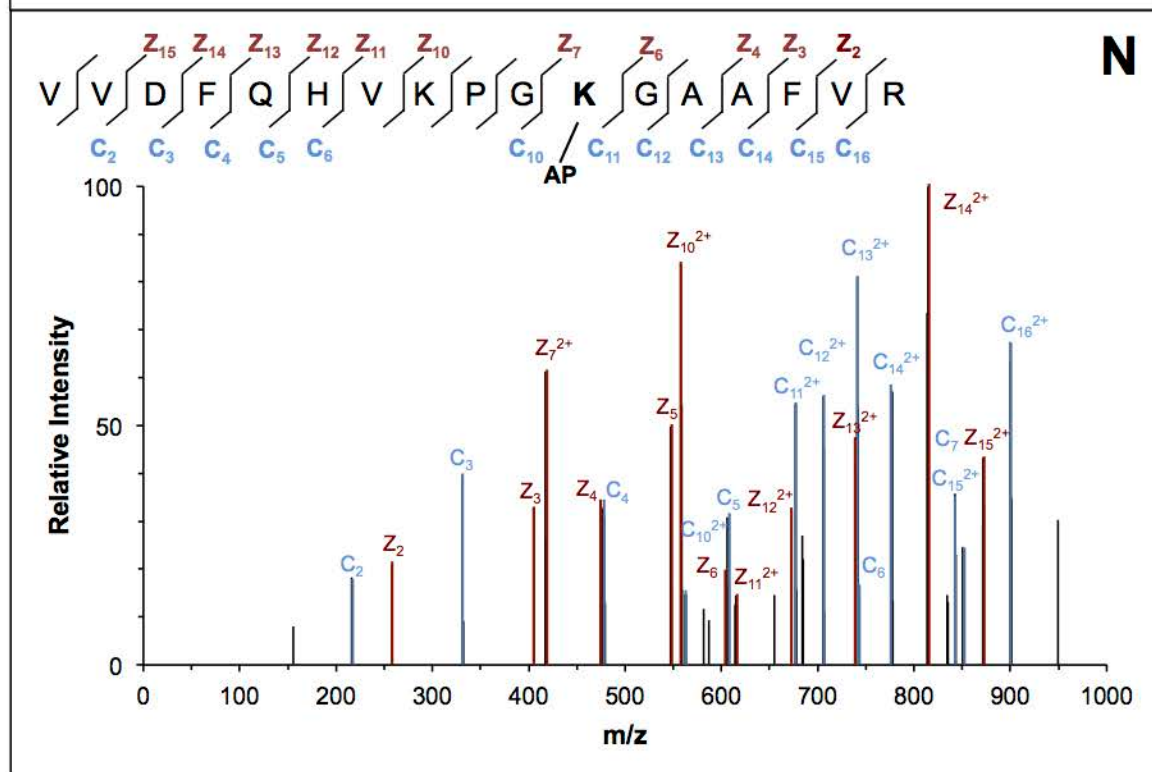

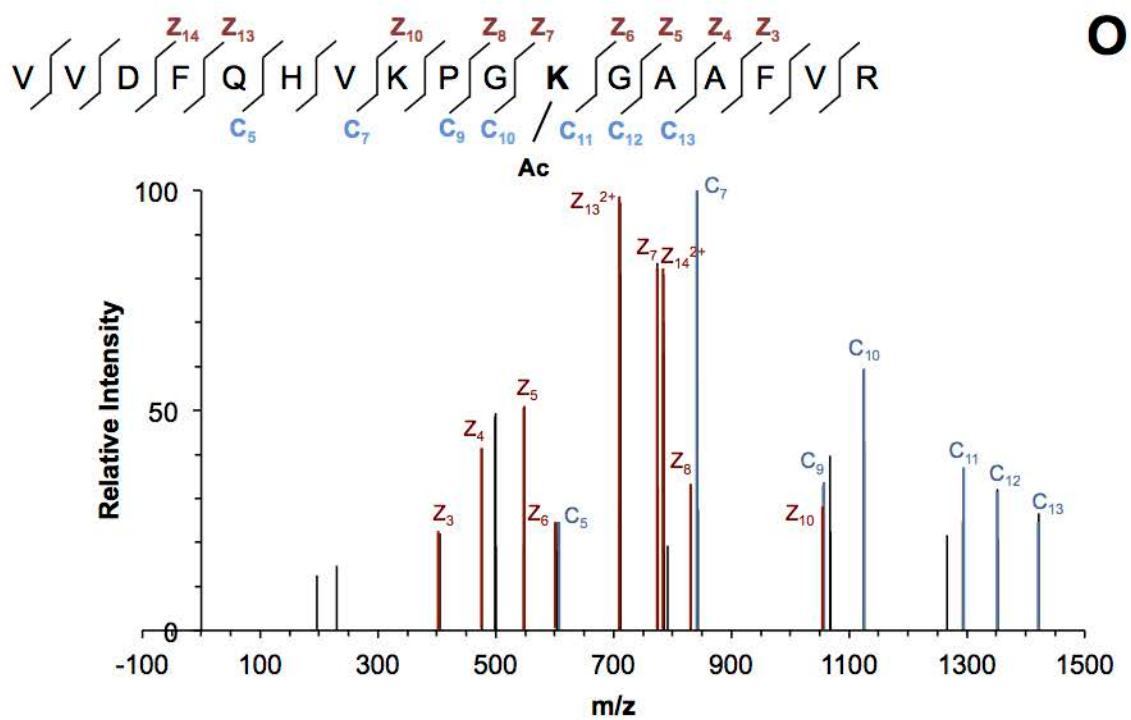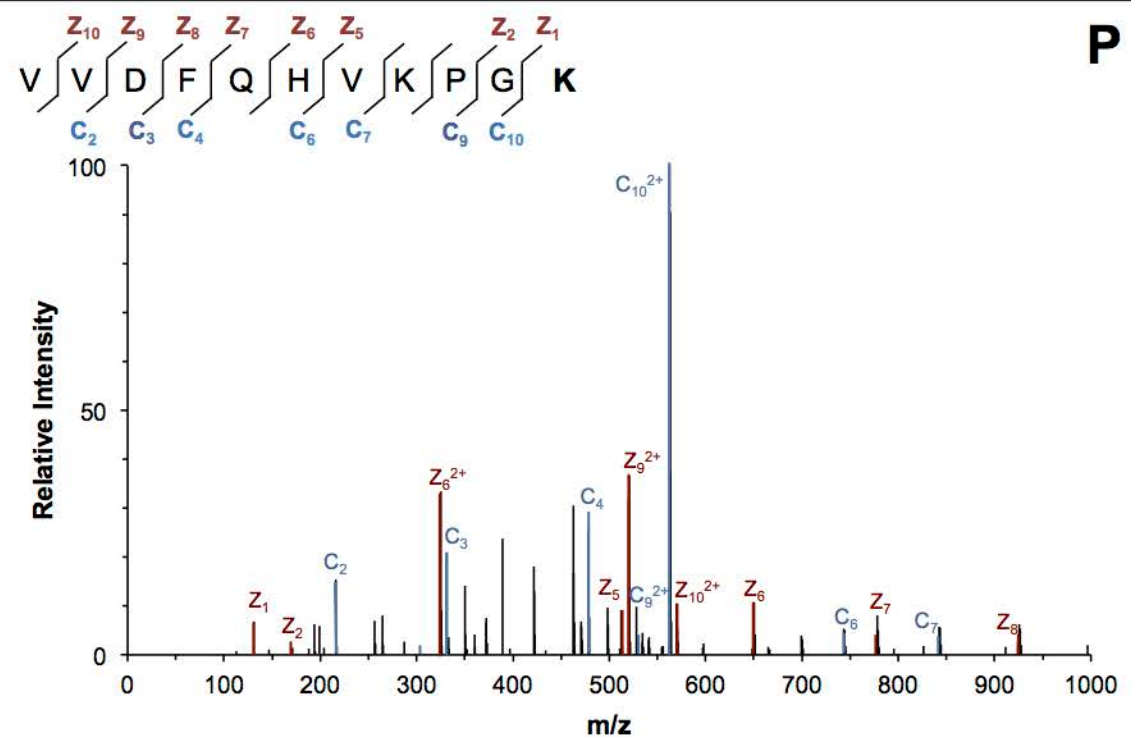

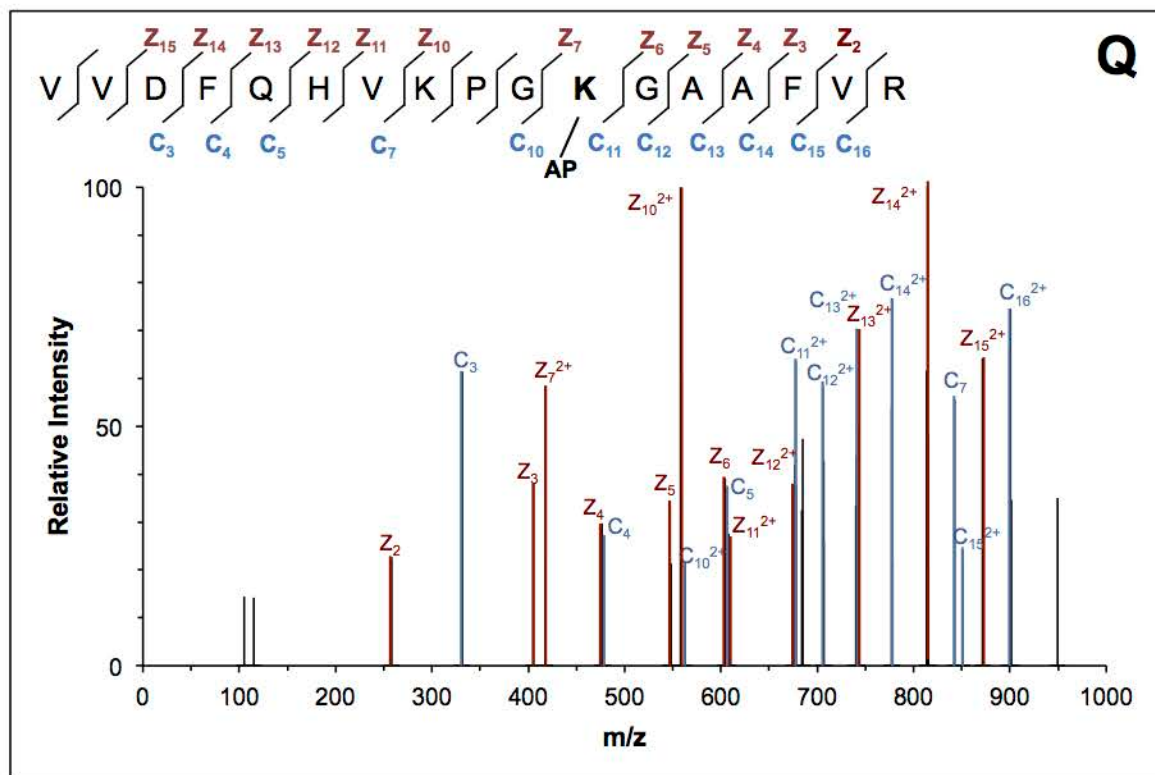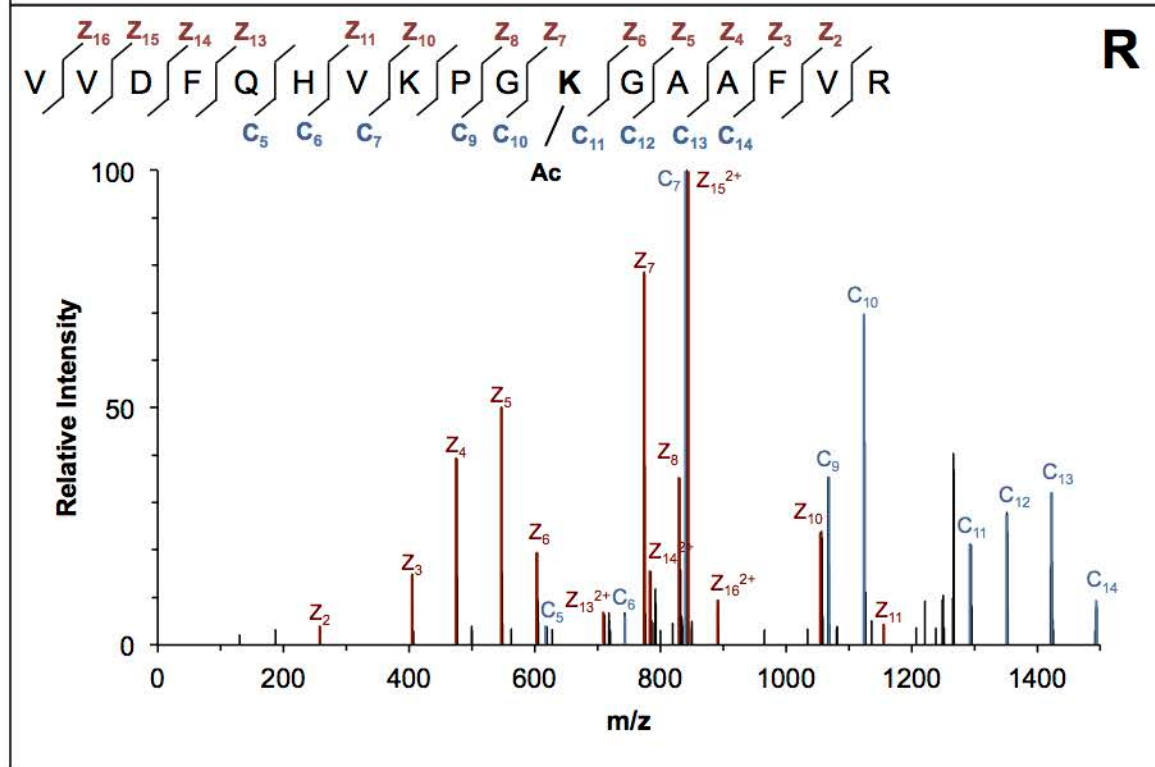

Supplement: FIG S3 [file mbo002183798sf3.pdf]

Figure S4

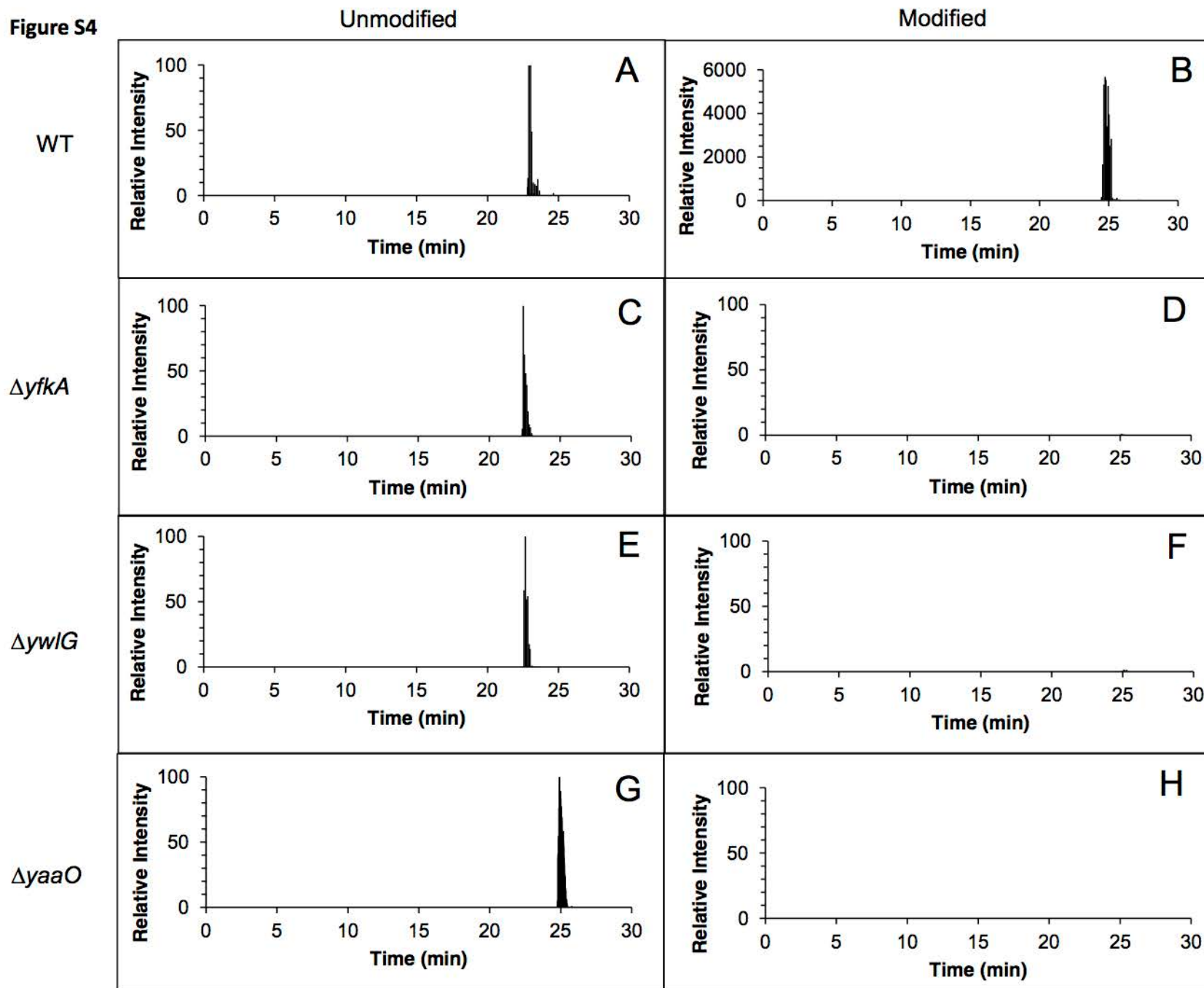

Supplement: FIG S4 [file mbo002183798sf4.pdf]

Figure S5

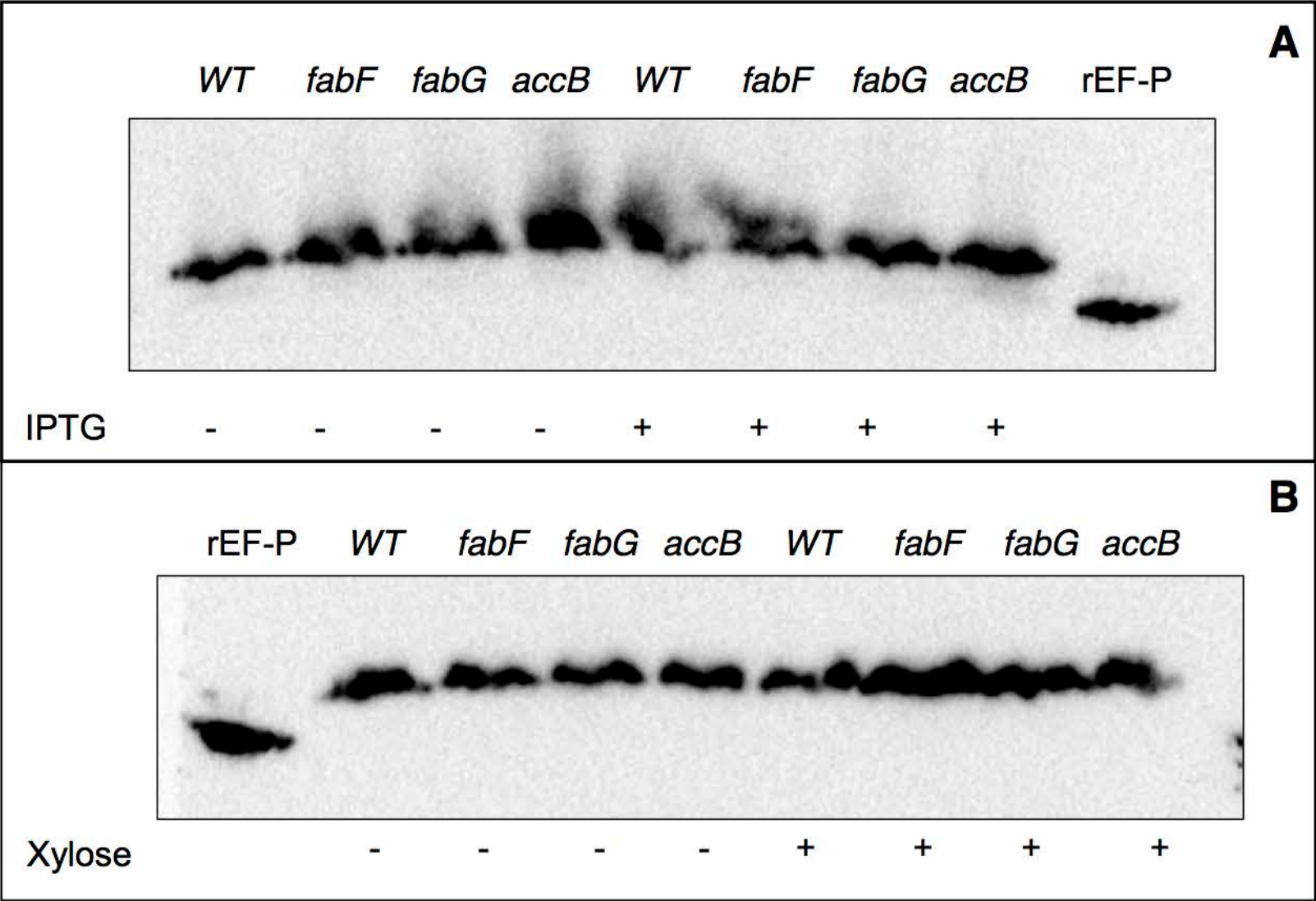

Supplement: FIG S5 [file mbo002183798sf5.pdf]
