# Supplementary material for: EF-P Posttranslational Modification Has Variable Impact on Polyproline Translation in Bacillus subtilis
Source: mBio. 2018 Apr 3;9(2):e00306-18. doi: 10.1128/mBio.00306-18 (PMC5885033; doi:10.1128/mBio.00306-18)
Supplement: TABLE S1 [file mbo002183798st1.pdf]

Table S1. Transposon insertion suppressors of *ymfI*

Strain	Insertion tag*
gsaB	
DK4374	TGATCTAGA
DK4367	TATATGATC
DK4375	TATTTGTTG
DK4304	TACATTTGC
DK4307	TACAGGCCG
DK4376	TAGCTATCG
DK4384	TACATAAAG
DK4372	TACATAAAG
DK4373	TATGTGCTG
yaaO	
DK4308	TATCGTGCC
DK4371	TATAAAGGT
DK4305	TATATAAAG
DK4300	TAAGAACCC
DK4366	TACATAAGC
DK4382	TATAGCCAG
DK4306	TATCTTCTG
***ynbAB* operon**	
ynbA	
DK4302	TATACTTCT
DK4365	TACATATAC
ynbB	
DK4370	TAATGTGTC
DK4369	TAAAGGAGC
DK4364	TATAGCCGA
DK4381	TATGAGCAA
DK4301	TAGGCTGGA
DK4383	TAATTCGAT
yfkA	
DK4303	TATTCACCG
ywlG	
DK4368	TAGATGCTT

*The insertion tag indicates the 9 basepairs immediately adjacent to the transposon insertion element upstream of the kanamycin resistance cassette.
