# Supplementary material for: EF-P Posttranslational Modification Has Variable Impact on Polyproline Translation in Bacillus subtilis
Source: mBio. 2018 Apr 3;9(2):e00306-18. doi: 10.1128/mBio.00306-18 (PMC5885033; doi:10.1128/mBio.00306-18)
Supplement: TABLE S2 [file mbo002183798st2.pdf]

Table S2. Phenotype microarray conditions where WT and Δ*efp* display differences in relative growth

Growth Condition (WT Enhanced Resipration)	PM	Mode
2,3-Butanone	2A	C-Source, Alcohol
Sodium Benzoate, pH 5.0	9	Toxicity, Benzoate
Sodium Sulfate	9	Osmotic Sensitivity
Sodium Formate	9	Osmotic Sensitivity
Urea	9	Osmotic Sensitivity
Sodium Nitrate	9	Toxicity
Sodium Nitrite	9	Toxicity
pH 4.5	10	pH
pH 4.5 + AA	10	pH, decarboxylase
pH 4.5 + Ornithine	10	pH, decarboxylase
pH 4.5 + Cysteic acid	10	pH, decarboxylase
pH 4.5 + Diamino-Pimelic Acid	10	pH, decarboxylase
pH 4.5 + Urea	10	pH, decarboxylase
Tobramycine	12B	Protein Synthesis, 30S
Nickel Chloride	13B	Toxic Cation
2, 2' Dipyridyl	13B	Chelator, Lipophilic
Potassium Chomate	13B	Toxic Cation
Oxolinic Acid	13B	DNA Topoisomerase
Doxycycline	13B	Protein Synthesis, 30S
Cesium Chloride	13B	Toxic Cation
Thallium (I) Acetate	13B	Toxic Cation
Cobalt Chloride	13B	Toxic Cation
Manganese (II) Chloride	13B	Toxic Cation
Cupric Chloride	13B	Toxic Cation
Acriflavine	14	DNA intercalator
Furaltadone	14	Nitro Compound
1-Hydroxypyridine-2-thione	14	Biofilm Inhibitor
Sodium Cyanate	14	Toxic Anion
Iodoacetate	14	Oxidizes Sulfhydryls
Sodium Dichromate	14	Toxic Anion
Cefoxitin	14	Wall
Sodium Metaborate	14	Toxic Anion
Carbenicillin	14	Wall
Nitrite	14	Toxic Anion
EDTA	15	Chelator, Hydrophilic
5,7-Dichloro-8-hydroxy-quinalone	15	Chelator, Lipophilic
5,7-Dichloro08-hydroxyquinoline	15	Chelator, Lipophilic
Nitrofurazone	15	Nitro Compound
Menadione	15	Respiration

2-Nitroimidazole	15	Nitro Compound
Norfloxacin	16	DNA topoisomerase
Protamine Sulfate	16	Membrane
Cinoxacin	16	DNA Topoisomerase
Streptomycin	16	Protein Synthesis, 30S
Sorbic Acid	16	Respiration
D-Serine	17	Inhibits 3PGA denydrogenase
Sodium salicylate	17	Biofilm Inhibitor
Aminotriazole	17	Inhibits Catalse, Histidine
Compound 48/80	17	cyclic AMP phosphodiesterase inhibitor
D,L-Methionine hydroxamate	17	tRNA synthetase
Phenylarsine Oxide	17	Tyrosine Phosphatase Inhibitor
Ketoprofen	18C	Biofilm Inhibitor
Pyrophosphate	18C	Chelator, Hydrophilic
Thiamphenicol	18C	Protein Synthesis, 30S
Sodium m-arsenite	18C	Toxic Anion
Sodium Bromate	18C	Toxic Anion
Lidocaine	18C	Ion Channel Inhibitor
Sodium Periodate	18C	Toxic Anion
Hexamminecobalt (III) Chloride	19	DNA Syntehsis
Ornidazole	20	Nitro Compound
Patulin	20	microtubulin polymerization inhibitor
**Growth Condition (Δ*efp* Enhanced Resipration)**	**PM**	**Mode**
D-Glucose-1-Phosphate	3	P-Source, Organic
Sulfadiazine	12B	Folate Antagonist, PABA analog
Sulfamethazine	12B	Folate Antagonist, PABA analog
Sulfathiazole	12B	Folate Antagonist, PABA analog
Sulfamethoxazole	12B	Folate Antagonist, PABA analog
Cefuroxime	13B	Wall, cephalosporin
Puromycin	15	Protein Synthesis, 30S
Sulfanilamide	16	Folate Antagonist, PABA analog
Trimethoprim	16	Folate Antagonist, PABA analog
1-Chloro-2,4-dinitrobenzene	16	Oxidizes sulfhydryls, depletes gluathione
Sulfachloropyridazine	17	Folate Antagonist, PABA analog
Sulfamonomethoxine	17	Folate Antagonist, PABA analog
Sulfisoxazole	17	Folate Antagonist, PABA analog
Plumbagin	18	Oxidizing Agent
