# Supplementary material for: EF-P Posttranslational Modification Has Variable Impact on Polyproline Translation in Bacillus subtilis
Source: mBio. 2018 Apr 3;9(2):e00306-18. doi: 10.1128/mBio.00306-18 (PMC5885033; doi:10.1128/mBio.00306-18)
Supplement: TABLE S3 [file mbo002183798st3.pdf]

Table S3: Strains

Strain	Genotype	Reference
3610	Wild type	
DK1042	*comI*^{Q12L}	Konkol, 2013
DK2050	*comI*^{Q12L} *Δefp*	Rajkovic, 2016
DK2448	*comI*^{Q12L} *Δefp amyE::*$P_{hyspank}$*-efp-flag spec*	Rajkovic, 2016
DK2886	*comI*^{Q12L} *Δefp ymfI::tet*	Hummels, 2017
DK3621	*comI*^{Q12L} *ymfI::tet*	Hummels, 2017
DK3780	*comI*^{Q12L} *Δefp amyE::*P_{yqhS}*-efp cat*	Hummels, 2017
DK3789	*comI*^{Q12L} *Δefp ymfI::tet amyE::*P_{yqhS}*-efp cat*	Hummels, 2017
DK3894	*comI*^{Q12L} *ΔyaaO*	
DK3908	*comI*^{Q12L} *ΔyaaO efp::tet amyE::* $P_{hyspank}$*-efp-flag spec*	
DK4077	*comI*^{Q12L} *ΔyaaO ymfI::tet*	
DK4300	*comI*^{Q12L} *Δefp ymfI::tet amyE::*P_{yqhS}*-efp cat yaaO::TnYLB kan*	
DK4301	*comI*^{Q12L} *Δefp ymfI::tet amyE::*P_{yqhS}*-efp cat ynbB::TnYLB kan*	
DK4302	*comI*^{Q12L} *Δefp ymfI::tet amyE::*P_{yqhS}*-efp cat ynbA::TnHyJump kan*	
DK4303	*comI*^{Q12L} *Δefp ymfI::tet amyE::*P_{yqhS}*-efp cat yfkA:: TnHyJump kan*	
DK4304	*comI*^{Q12L} *Δefp ymfI::tet amyE::*P_{yqhS}*-efp cat gsaB:: TnHyJump kan*	
DK4305	*comI*^{Q12L} *Δefp ymfI::tet amyE::*P_{yqhS}*-efp cat yaaO:: TnHyJump kan*	
DK4306	*comI*^{Q12L} *Δefp ymfI::tet amyE::*P_{yqhS}*-efp cat yaaO:: TnHyJump kan*	
DK4307	*comI*^{Q12L} *Δefp ymfI::tet amyE::*P_{yqhS}*-efp cat gsaB:: TnHyJump kan*	
DK4308	*comI*^{Q12L} *Δefp ymfI::tet amyE::*P_{yqhS}*-efp cat yaaO:: TnHyJump kan*	
DK4310	*comI*^{Q12L} *Δefp amyE::*$P_{hyspank}$*-efp-flag spec ynbB::TnYLB kan*	
DK4313	*comI*^{Q12L} *Δefp amyE::*$P_{hyspank}$*-efp-flag spec gsaB::TnHyJump kan*	
DK4364	*comI*^{Q12L} *Δefp ymfI::tet amyE::*P_{yqhS}*-efp cat ynbB::TnYLB kan*	
DK4365	*comI*^{Q12L} *Δefp ymfI::tet amyE::*P_{yqhS}*-efp cat ynbA::TnYLB kan*	
DK4366	*comI*^{Q12L} *Δefp ymfI::tet amyE::*P_{yqhS}*-efp cat yaaO::TnYLB kan*	
DK4367	*comI*^{Q12L} *Δefp ymfI::tet amyE::*P_{yqhS}*-efp cat gsaB::TnYLB kan*	
DK4368	*comI*^{Q12L} *Δefp ymfI::tet amyE::*P_{yqhS}*-efp cat ywlG::TnYLB kan*	
DK4369	*comI*^{Q12L} *Δefp ymfI::tet amyE::*P_{yqhS}*-efp cat ynbB::TnYLB kan*	
DK4370	*comI*^{Q12L} *Δefp ymfI::tet amyE::*P_{yqhS}*-efp cat ynbB::TnYLB kan*	
DK4371	*comI*^{Q12L} *Δefp ymfI::tet amyE::*P_{yqhS}*-efp cat yaaO::TnYLB kan*	
DK4372	*comI*^{Q12L} *Δefp ymfI::tet amyE::*P_{yqhS}*-efp cat gsaB::TnYLB kan*	
DK4373	*comI*^{Q12L} *Δefp ymfI::tet amyE::*P_{yqhS}*-efp cat gsaB::TnYLB kan*	
DK4374	*comI*^{Q12L} *Δefp ymfI::tet amyE::*P_{yqhS}*-efp cat gsaB::TnYLB kan*	
DK4375	*comI*^{Q12L} *Δefp ymfI::tet amyE::*P_{yqhS}*-efp cat gsaB::TnYLB kan*	
DK4376	*comI*^{Q12L} *Δefp ymfI::tet amyE::*P_{yqhS}*-efp cat gsaB::TnYLB kan*	
DK4381	*comI*^{Q12L} *Δefp ymfI::tet amyE::*P_{yqhS}*-efp cat ynbB::TnYLB kan*	
DK4382	*comI*^{Q12L} *Δefp ymfI::tet amyE::*P_{yqhS}*-efp cat yaaO::TnYLB kan*	
DK4383	*comI*^{Q12L} *Δefp ymfI::tet amyE::*P_{yqhS}*-efp cat ynbB::TnYLB kan*	
DK4384	*comI*^{Q12L} *Δefp ymfI::tet amyE::*P_{yqhS}*-efp cat gsaB::TnHyJump kan*	
DK4564	*comI*^{Q12L} *ΔyfkA*	
DK4572	*comI*^{Q12L} *ΔyfkA efp::tet amyE::*$P_{hyspank}$*-efp-flag spec*	
DK4573	*comI*^{Q12L} *Δefp amyE::*$P_{hyspank}$*-efp-flag spec ywlG::TnYLB kan*	
DK4601	*comI*^{Q12L} *ΔgsaB*	
DK4604	*comI*^{Q12L} *ΔynbB*	
DK4605	*comI*^{Q12L} *ΔynbA*	
DK4612	*comI*^{Q12L} *ΔywlG*	
DK4815	*comI*^{Q12L} *ΔgsaB ΔyaaO efp::tet amyE::*$P_{hyspank}$*-efp-flag spec*	
DK5170	*comI*^{Q12L} *ΔyfkA ymfI::tet*	
DK5171	*comI*^{Q12L} *ΔgsaB ymfI::tet*	
DK5172	*comI*^{Q12L} *ΔynbB ymfI::tet*	
DK5173	*comI*^{Q12L} *ΔynbA ymfI::tet*	
DK5174	*comI*^{Q12L} *ΔywlG ymfI::tet*	

DK5165	comI^{Q12L} ΔyfkA amyE::P_{yfkA}-yfkA spec	
DK5166	comI^{Q12L} ΔywlG amyE::P_{ywlF}-ywlG spec	
DK5167	comI^{Q12L} ΔyaaO amyE::P_{xpcA}-yaaO spec	
DK5304	comI^{Q12L} ΔyfkA ymfI::tet amyE::P_{yfkA}-yfkA spec	
DK5305	comI^{Q12L} ΔyaaO ymfI::tet amyE::P_{xpcA}-yaaO spec	
DK5308	comI^{Q12L} ΔgsaB amyE::P_{gsaB}-gsaB spec	
DK5309	comI^{Q12L} ΔynbB amyE::P_{ynbA}-ynbB spec	
DK5310	comI^{Q12L} ΔynbA amyE::P_{ynbA}-ynbA spec	
DK5320	comI^{Q12L} ΔgsaB ymfI::tet amyE::P_{gsaB}-gsaB spec	
DK5321	comI^{Q12L} ΔynbB ymfI::tet amyE::P_{ynbA}-ynbB spec	
DK5322	comI^{Q12L} ΔynbA ymfI::tet amyE::P_{ynbA}-ynbA spec	
DK5323	comI^{Q12L} ymfI::tet ynbA::TnYLB kan	
DK5326	comI^{Q12L} ΔywlG ymfI::tet amyE::P_{ywlF}-ywlG spec	
DK5327	comI^{Q12L} ΔyaaO amyE::$P_{hyspank}$-yaaO spec	
DK5328	comI^{Q12L} ΔyaaO ymfI::tet amyE::$P_{hyspank}$-yaaO spec	
DK5339	comI^{Q12L} ymfI::tet ynbA::TnHyJump kan	
DK5340	comI^{Q12L} ymfI::tet ynbA::TnHyJump kan amyE::P_{ynbA}-ynbB spec	
DK5342	comI^{Q12L} ymfI::tet ynbA::TnYLB kan amyE::P_{ynbA}-ynbB spec	
DS235	ymfI::tet	Kearns, 2004
DS354	efp::tet	Kearns, 2004
DS2197	sfp^{0} swrA pMarA kan mls	Le Breton, 2006
DS7853	sfp^{0} swrA pTnHyJump kan mls	Pozsgai, 2012
AW112	comI^{Q12L} amyE::$P_{hyspank}$-gfp	
AW113	comI^{Q12L} amyE::$P_{hyspank}$-ppw-gfp	
AW114	comI^{Q12L} Δefp amyE::$P_{hyspank}$-gfp	
AW115	comI^{Q12L} Δefp amyE::$P_{hyspank}$-ppw-gfp	
AW118	comI^{Q12L} ΔgsaB amyE::$P_{hyspank}$-gfp	
AW119	comI^{Q12L} ΔgsaB amyE::$P_{hyspank}$-ppw-gfp	
AW120	comI^{Q12L} ΔynbB amyE::$P_{hyspank}$-gfp	
AW121	comI^{Q12L} ΔynbB amyE::$P_{hyspank}$-ppw-gfp	
AW122	comI^{Q12L} ΔyfkA amyE::$P_{hyspank}$-gfp	
AW123	comI^{Q12L} ΔyfkA amyE::$P_{hyspank}$-ppw-gfp	
AW124	comI^{Q12L} ΔywlG amyE::$P_{hyspank}$-gfp	
AW125	comI^{Q12L} ΔywlG amyE::$P_{hyspank}$-ppw-gfp	
AW126	comI^{Q12L} amyE::spec	
AW146	comI^{Q12L} amyE:: $P_{hyspank}$-fabF	
AW147	comI^{Q12L} amyE:: $P_{hyspank}$-fabG	
AW148	comI^{Q12L} amyE:: $P_{hyspank}$-accB	
AW149	comI^{Q12L} amyE::$P_{hyspank}$-ppp-gfp	
AW150	comI^{Q12L} Δefp amyE::$P_{hyspank}$-ppp-gfp	
AW156	comI^{Q12L} ymfI::tet amyE::$P_{hyspank}$-ppp-gfp	
AW157	comI^{Q12L} ΔyaaO amyE::$P_{hyspank}$-ppp-gfp	
AW158	comI^{Q12L} ΔgsaB amyE::$P_{hyspank}$-ppp-gfp	
AW159	comI^{Q12L} ΔynbB amyE::$P_{hyspank}$-ppp-gfp	
AW160	comI^{Q12L} ΔyfkA amyE::$P_{hyspank}$-ppp-gfp	
AW161	comI^{Q12L} ΔywlG amyE::$P_{hyspank}$-ppp-gfp	
AW163	comI^{Q12L} amyE::$P_{hyspank}$-ppe-gfp	
AW164	comI^{Q12L} Δefp amyE::$P_{hyspank}$-ppe-gfp	
AW165	comI^{Q12L} ymfI::tet amyE::$P_{hyspank}$-ppe-gfp	
AW166	comI^{Q12L} ΔyaaO amyE::$P_{hyspank}$-ppe-gfp	
AW167	comI^{Q12L} ΔgsaB amyE::$P_{hyspank}$-ppe-gfp	
AW168	comI^{Q12L} ΔynbB amyE::$P_{hyspank}$-ppe-gfp	
AW169	comI^{Q12L} ΔyfkA amyE::$P_{hyspank}$-ppe-gfp	
AW170	comI^{Q12L} ΔywlG amyE::$P_{hyspank}$-ppe-gfp	
RT01	comI^{Q12L} ymfI::tet amyE::$P_{hyspank}$-gfp	

RT02	*comI*^{Q12L} *ymfI::tet amyE::*$P_{hyspank}$*-ppw-gfp*
RT03	*comI*$^{Q12\ L}$ Δ*yaaO amyE::*$P_{hyspank}$*-gfp*
RT04	*comI*^{Q12L} Δ*yaaO amyE::*$P_{hyspank}$*-ppw-gfp*
BEC11340	*lacA::*P_{xyl}*-dcas9 amyE::*P_{veg}*-sgRNA(fabF)*
BEC15910	*lacA::*P_{xyl}*-dcas9 amyE::*P_{veg}*-sgRNA(fabG)*
BEC24350	*lacA::*P_{xyl}*-dcas9 amyE::*P_{veg}*-sgRNA(accB)*
