# Supplementary material for: EF-P Posttranslational Modification Has Variable Impact on Polyproline Translation in Bacillus subtilis
Source: mBio. 2018 Apr 3;9(2):e00306-18. doi: 10.1128/mBio.00306-18 (PMC5885033; doi:10.1128/mBio.00306-18)
Supplement: TABLE S4 [file mbo002183798st4.pdf]

Table S4: Plasmids

Plasmid	Genotype	Reference
pAH25	*amyE spec amp*	Gift from Amy Camp, Mt. Holyoke College
pDR111	*amyE::*$P_{hyspank}$ *spec amp*	Gift from David Rudner, Harvard Medical School
pMimiMAD	*ori*$P^{t.s}$ *mls amp*	(Patrick and Kearns, 2008)
pKRH58	*ΩΔyaaO mls amp*	
pKRH69	*ΩΔyfkA mls amp*	
pKRH70	*ΩΔynbB mls amp*	
pKRH71	*ΩΔynbA mls amp*	
pKRH72	*ΩΔgsaB mls amp*	
pKRH73	*ΩΔywlG mls amp*	
pKRH86	*amyE::*P_{ynbA}*-ynbB spec amp*	
pKRH87	*amyE::*P_{yfkA}*-yfkA spec amp*	
pKRH88	*amyE::*P_{ywlF}*-ywlG spec amp*	
pKRH89	*amyE::*P_{xpcA}*-yaaO spec amp*	
pKRH90	*amyE::*P_{gsaB}*-gsaB spec amp*	
pKRH101	*amyE::*P_{ynbA}*-ynbA spec amp*	
pKRH112	*amyE::*$P_{hyspank}$*-yaaO spec amp*	
pAW40	*amyE::*$P_{hyspank}$*-ppw-gfp spec amp*	Rajkovic, 2016
pAW92	*amyE::*$P_{hyspank}$*-gfp spec amp*	Rajkovic, 2016
pAW93	*amyE::*$P_{hyspank}$*-ppp-gfp spec amp*	Rajkovic, 2016
pAW162	*amyE::*$P_{hyspank}$*-ppe-gfp spec amp*	
pAW143	*amyE::*$P_{hyspank}$*-fabF spec amp*	
pAW144	*amyE::*$P_{hyspank}$*-fabG spec amp*	
pAW145	*amyE::*$P_{hyspank}$*-accB spec amp*	
