# Supplementary material for: EF-P Posttranslational Modification Has Variable Impact on Polyproline Translation in Bacillus subtilis
Source: mBio. 2018 Apr 3;9(2):e00306-18. doi: 10.1128/mBio.00306-18 (PMC5885033; doi:10.1128/mBio.00306-18)
Supplement: TABLE S5 [file mbo002183798st5.pdf]

Table S5: Primers

Primer	Sequence
695	GCTTGTAAATTCTATCATAATTG
696	AGGGAATCATTTGAAGGTTGG
1657	TTAAGCTTAGTCGACAGTAAGGAGGAACTACTATGAGTAAACCGCCGGAAAGAGAAGAACTTTTCACTGG
2567	GTACATCCGCAACTGTCCATA
2818	TCTCCCAATCAGGCTTGA
4933	GTCGACTCTAGAGGATCCCCGAAGCAACAGGCTGAATCGA
4934	TTCATATTCCCTTGAACATGCGTTCTCGCATGTTGAATTAATGCTT
4935	AAGCATTAATTCAACATGCGAGAACGCATGTTCAAGGGAATATGAA
4936	GTGAATTCGAGCTCGGTACCCATCCTGAACGACGAGGTCTT
5279	GTCGACTCTAGAGGATCCCCATAATATGGCCCTCATCTGAC
5280	TTACGCCCGAGTTTGACCCTTATTATCAGGATACAGACTTCAC
5281	GTGAAGTCTGTATCCTGATAATAAGGGTCAAACTCGGGCGTA
5282	GTGAATTCGAGCTCGGTACCCGGTAGAGCTGGCGAACGAG
5283	GTCGACTCTAGAGGATCCCCCCTGCCAAGCGGCTACAG
5284	CAATGAATCCACTGCACTGCACTTCAATAATTCTCCGTGTGTT
5285	AACACACGGAGAATTATTGAAGTGCAGTGCAGTGGATTCATTG
5286	GTGAATTCGAGCTCGGTACCCGAAGCGGGACATGTCGCC
5287	GTCGACTCTAGAGGATCCCCTATATCGGACACACCGCCC
5288	ATAGCCGGATATGTCATACTGATGCGGCAGCTGACATCCG
5289	CGGATGTCAGCTGCCGCATCAGTATGACATATCCGGCTAT
5290	GTGAATTCGAGCTCGGTACCCAATGAGAGATCCCGCCATCA
5291	GTCGACTCTAGAGGATCCCCGAAGCGCTTCGGCTACATG
5292	TATGTGCTGTGGTAATAAACCAACCGCCGACAATGTGCTGT
5293	ACAGCACATTGTCGGCGGTTGGTTTATTACCACAGCACATA
5294	GTGAATTCGAGCTCGGTACCCGTCGTCACCGGCAGATTCT
5295	GTCGACTCTAGAGGATCCCCGAAAGTAGCCATTGCATCGG
5296	CGGCCGTGTACGCGCCAATTGGTCCTGAAACTCAGACAG
5297	CTGTCTGAGTTTCAGGACCAATTGGCGCGTACACGGCCG
5298	GTGAATTCGAGCTCGGTACCCATTTCCTTCGCGCGGTCAC
5877	AGGAGGAAGCTTGATACTCGCCGGATATTCAC
5878	TCCTCGCTAGCTGCATATTGGGCAGTAGTATTA
5879	AGGAGGCTAGCTCGGTGAATTAAAGAAGTATATG
5880	TCCTCGCATGCGATGTAAGGATTCTTAACATCAA
5881	AGGAGGGATCCTCCAGGTCATCAAGCATGCT
5882	AGGAGGGATCCTCCAGGTCATCAAGCATGCT
5883	TCCTCGCTAGCATCTCCGTAAGTGTTTCTTTCA
5884	AGGAGAAGCTTTGAATGTCAATGTCCGCTCG
5885	TCCTCGCTAGCTTCCTCCCAGCGTGTGTGA
5886	AGGAGGCTAGCGCGTATTGGAAAAATCTCCGA
5887	TCCTCGAATTCAAGTTCGGAATTTATTTCACTATCT
5888	AGGAGAAGCTTTCGAGAAAATCAAACAAATACAAA
5889	TCCTCGCTAGCTGTGTCTATACTCAGTTTTATTAT
5909	TCCTCGCTAGCGGGATCGATACAAATATTCAAG
5945	AGGAGGCATGCGTGTTAATGTGTCAAACATGTC
5946	AGGAGGGATCCGAAAATCGCATGCTTCAGTTGT
6102	ATTAAGCTTAGTCGACAGTAAGGAGGAACTACTATGACTAAAAAAAGAGTAGTTGTTACAGGACTTG
6103	CGAATTAGCTTGCATGTTATGATTGATATTTTTTAAAGATTAATGTTGCGTTGTG
6104	ATTAAGCTTAGTCGACAGTAAGGAGGAACTACTATGCAAAGTTTACAACATAAAACTGCACTAATC
6105	CGAATTAGCTTGCATGTTAAGGATTTGTTGACCATAATCCCGCTG
6106	AATTAAGCTTAGTCGACAGTAAGGAGGAACTACTATGTTAAATATCAAAGAAATCCACGAGCTG
6107	CGAATTAGCTTGCATGTTACTCCGCTTTTACAAGAAATAGAGGTTGTC
7514	CCGAATTAGCTTGCATGTTAATTTGTAGAGCTCATCCATGCCATGTG
